# Convergence Rates for Empirical Estimation of Binary Classification Bounds

**DOI:** 10.3390/e21121144

**Published:** 2019-11-23

**Authors:** Salimeh Yasaei Sekeh, Morteza Noshad, Kevin R. Moon, Alfred O. Hero

**Affiliations:** 1 School of Computing and Information Science, University of Maine, Orono, ME 04469, USA; 2 Department of Electrical Engineering and Computer Science, University of Michigan, Ann Arbor, MI 48109, USA; noshad@umich.edu (M.N.);; 3 Department of Mathematics and Statistics, Utah State University, Logan, UT 84322, USA; kevin.moon@usu.edu

**Keywords:** classification, Bayes error rate, Henze–Penrose divergence, Friedman–Rafsky test statistic, convergence rates, bias and variance trade-off, concentration bounds, minimal spanning trees

## Abstract

Bounding the best achievable error probability for binary classification problems is relevant to many applications including machine learning, signal processing, and information theory. Many bounds on the Bayes binary classification error rate depend on information divergences between the pair of class distributions. Recently, the Henze–Penrose (HP) divergence has been proposed for bounding classification error probability. We consider the problem of empirically estimating the HP-divergence from random samples. We derive a bound on the convergence rate for the Friedman–Rafsky (FR) estimator of the HP-divergence, which is related to a multivariate runs statistic for testing between two distributions. The FR estimator is derived from a multicolored Euclidean minimal spanning tree (MST) that spans the merged samples. We obtain a concentration inequality for the Friedman–Rafsky estimator of the Henze–Penrose divergence. We validate our results experimentally and illustrate their application to real datasets.

## 1. Introduction

Divergence measures between probability density functions are used in many signal processing applications including classification, segmentation, source separation, and clustering (see [1,2,3]). For more applications of divergence measures, we refer to [4].

In classification problems, the Bayes error rate is the expected risk for the Bayes classifier, which assigns a given feature vector x to the class with the highest posterior probability. The Bayes error rate is the lowest possible error rate of any classifier for a particular joint distribution. Mathematically, let $x_{1},x_{2},\ldots ,x_{N}\in R^{d}$ be realizations of random vector X and class labels S∈{0,1}, with prior probabilities p=P(S=0) and q=P(S=1), such that p+q=1. Given conditional probability densities $f_{0}\left(x\right)$ and $f_{1}\left(x\right)$, the Bayes error rate is given by

(1) $$\epsilon =\int_{R^{d}}\min\left\{pf_{0}\left(x\right),qf_{1}\left(x\right)\right\}dx.$$

The Bayes error rate provides a measure of classification difficulty. Thus, when known, the Bayes error rate can be used to guide the user in the choice of classifier and tuning parameter selection. In practice, the Bayes error is rarely known and must be estimated from data. Estimation of the Bayes error rate is difficult due to the nonsmooth min function within the integral in (1). Thus, research has focused on deriving tight bounds on the Bayes error rate based on smooth relaxations of the min function. Many of these bounds can be expressed in terms of divergence measures such as the Bhattacharyya [5] and Jensen–Shannon [6]. Tighter bounds on the Bayes error rate can be obtained using an important divergence measure known as the Henze–Penrose (HP) divergence [7,8].

Many techniques have been developed for estimating divergence measures. These methods can be broadly classified into two categories: (i) plug-in estimators in which we estimate the probability densities and then plug them in the divergence function [9,10,11,12], (ii) entropic graph approaches, in which the relationship between the divergence function and a graph functional in Euclidean space is derived [8,13]. Examples of plug-in methods include k-nearest neighbor (K-NN) and Kernel density estimator (KDE) divergence estimators. Examples of entropic graph approaches include methods based on minimal spanning trees (MST), K-nearest neighbors graphs (K-NNG), minimal matching graphs (MMG), traveling salesman problem (TSP), and their power-weighted variants.

Disadvantages of plug-in estimators are that these methods often require assumptions on the support set boundary and are more computationally complex than direct graph-based approaches. Thus, for practical and computational reasons, the asymptotic behavior of entropic graph approaches has been of great interest. Asymptotic analysis has been used to justify graph based approaches. For instance, in [14], the authors showed that a cross match statistic based on optimal weighted matching converges to the the HP-divergence. In [15], a more complex approach based on the K-NNG was proposed that also converges to the HP-divergence.

The first contribution of our paper is that we obtain a bound on the convergence rates for the Friedman and Rafsky (FR) estimator of the HP-divergence, which is based on a multivariate extension of the non-parametric run length test of equality of distributions. This estimator is constructed using a multicolored MST on the labeled training set where MST edges connecting samples with dichotomous labels are colored differently from edges connecting identically labeled samples. While previous works have investigated the FR test statistic in the context of estimating the HP-divergence (see [8,16]), to the best of our knowledge, its minimax MSE convergence rate has not been previously derived. The bound on convergence rate is established by using the umbrella theorem of [17], for which we define a dual version of the multicolor MST. The proposed dual MST in this work is different than the standard dual MST introduced by Yukich in [17]. We show that the bias rate of the FR estimator is bounded by a function of *N*, η and *d*, as $O\left({\left(N\right)}^{-\eta^{2}/\left(d\left(\eta +1\right)\right)}\right)$, where *N* is the total sample size, *d* is the dimension of the data samples d≥2, and η is the Hölder smoothness parameter 0<η≤1. We also obtain the variance rate bound as $O\left({\left(N\right)}^{-1}\right)$.

The second contribution of our paper is a new concentration bound for the FR test statistic. The bound is obtained by establishing a growth bound and a smoothness condition for the multicolored MST. Since the FR test statistic is not a Euclidean functional, we cannot use the standard subadditivity and superadditivity approaches of [17,18,19]. Our concentration inequality is derived using a different Hamming distance approach and a dual graph to the multicolored MST.

We experimentally validate our theoretic results. We compare the MSE theory and simulation in three experiments with various dimensions d=2,4,8. We observe that, in all three experiments, as sample size increases, the MSE rate decreases and, for higher dimensions, the rate is slower. In all sets of experiments, our theory matches the experimental results. Furthermore, we illustrate the application of our results on estimation of the Bayes error rate on three real datasets.

### 1.1. Related Work

Much research on minimal graphs has focused on the use of Euclidean functionals for signal processing and statistics applications such as image registration [20,21], pattern matching [22], and non-parametric divergence estimation [23]. A K-NNG-based estimator of Rényi and *f*-divergence measures has been proposed in [13]. Additional examples of direct estimators of divergence measures include statistic based on the nonparametric two sample problem, the Smirnov maximum deviation test [24], and the Wald–Wolfowitz [25] runs test, which have been studied in [26].

Many entropic graph estimators such as MST, K-NNG, MMG, and TSP have been considered for multivariate data from a single probability density *f*. In particular, the normalized weight function of graph constructions all converge almost surely to the Rényi entropy of *f* [17,27]. For *N* uniformly distributed points, the MSE is $O(N^{-1/d})$ [28,29]. Later, Hero et al. [30,31] reported bounds on $L_{\gamma }$-norm bias convergence rates of power-weighted Euclidean weight functionals of order γ for densities *f* belonging to the space of Hölder continuous functions $\Sigma_{d}\left(\eta ,K\right)$ as $O\left(N^{-\alpha \eta /(\alpha \eta +1)\;1/d}\right)$, where 0<η≤1, d≥1, γ∈(1,d), and α=(d−γ)/d. In this work, we derive a bound on convergence rate of FR estimator for the HP-divergence when the density functions belong to the Hölder class, $\Sigma_{d}\left(\eta ,K\right)$, for 0<η≤1, d≥2 [32]. Note that throughout the paper we assume the density functions are absolutely continuous and bounded with support on the unit cube ${\left[0,1\right]}^{d}$.

In [28], Yukich introduced the general framework of continuous and quasi-additive Euclidean functionals. This has led to many convergence rate bounds of entropic graph divergence estimators.

The framework of [28] is as follows: Let *F* be finite subset of points in ${\left[0,1\right]}^{d}$, d≥2, drawn from an underlying density. A real-valued function $L_{\gamma }$ defined on *F* is called a Euclidean functional of order γ if it is of the form $L_{\gamma }\left(F\right)=\min_{E\in E}\sum_{e\in E}{\left|e\left(F\right)\right|}^{\gamma }$, where E is a set of graphs, *e* is an edge in the graph *E*, |e| is the Euclidean length of *e*, and γ is called the edge exponent or power-weighting constant. The MST, TSP, and MMG are some examples for which γ=1.

Following this framework, we show that the FR test statistic satisfies the required continuity and quasi-additivity properties to obtain similar convergence rates to those predicted in [28]. What distinguishes our work from previous work is that the count of dichotomous edges in the multicolored MST is not Euclidean. Therefore, the results in [17,27,30,31] are not directly applicable.

Using the isoperimetric approach, Talagrand [33] showed that, when the Euclidean functional $L_{\gamma }$ is based on the MST or TSP, then the functional $L_{\gamma }$ for derived random vertices uniformly distributed in a hypercube ${\left[0,1\right]}^{d}$ is concentrated around its mean. Namely, with high probability, the functional $L_{\gamma }$ and its mean do not differ by more than $C{\left(N\log N\right)}^{(d-\gamma )/2d}$. In this paper, we establish concentration bounds for the FR statistic: with high probability 1−δ, the FR statistic differs from its mean by not more than $O\left({\left(N\right)}^{(d-1)/d}{\left(\log(C/\delta )\right)}^{(d-1)/d}\right)$, where *C* is a function of *N* and *d*.

### 1.2. Organization

This paper is organized as follows. In Section 2, we first introduce the HP-divergence and the FR multivariate test statistic. We then present the bias and variance rates of the FR-based estimator of HP-divergence followed by the concentration bounds and the minimax MSE convergence rate. Section 3 provides simulations that validate the theory. All proofs and relevant lemmas are given in the Appendix A, Appendix B, Appendix C, Appendix D and Appendix E.

Throughout the paper, we denote expectation by E and variance by abbreviation Var. Bold face type indicates random variables. In this paper, when we say number of samples we mean number of observations.

## 2. The Henze–Penrose Divergence Measure

Consider parameters p∈(0,1) and q=1−p. We focus on estimating the HP-divergence measure between distributions $f_{0}$ and $f_{1}$ with domain $R^{d}$ defined by

(2) $$D_{p}\left(f_{0},f_{1}\right)=\frac{1}{4pq}\left[\int \frac{{\left(pf_{0}\left(x\right)-qf_{1}\left(x\right)\right)}^{2}}{pf_{0}\left(x\right)+qf_{1}\left(x\right)}\;dx-{\left(p-q\right)}^{2}\right].$$

It can be verified that this measure is bounded between 0 and 1 and, if $f_{0}\left(x\right)=f_{1}\left(x\right)$, then $D_{p}=0$. In contrast with some other divergences such as the Kullback–Liebler [34] and Rényi divergences [35], the HP-divergence is symmetrical, i.e., $D_{p}\left(f_{0},f_{1}\right)=D_{q}\left(f_{1},f_{0}\right)$. By invoking relation (3) in [8],

$$\begin{array}{c} \int \frac{{\left(pf_{0}\left(x\right)-qf_{1}\left(x\right)\right)}^{2}}{pf_{0}\left(x\right)+qf_{1}\left(x\right)}\;dx=1-4pqA_{p}\left(f_{0},f_{1}\right), \end{array}$$

where

$$\begin{array}{c} \begin{array}{ccc} A_{p}\left(f_{0},f_{1}\right) & = & \int \frac{f_{0}\left(x\right)f_{1}\left(x\right)}{pf_{0}\left(x\right)+qf_{1}\left(x\right)}\;dx=E_{f_{0}}\left[{\left(p\;\frac{f_{0}\left(X\right)}{f_{1}\left(X\right)}+q\right)}^{-1}\right],\\ u_{p}\left(f_{0},f_{1}\right) & = & 1-4pq\;A_{p}\left(f_{0},f_{1}\right), \end{array} \end{array}$$

one can rewrite $D_{p}$ in the alternative form:

$$\begin{array}{c} D_{p}\left(f_{0},f_{1}\right)=1-A_{p}\left(f_{0},f_{1}\right)=\frac{u_{p}\left(f_{0},f_{1}\right)}{4pq}-\frac{{\left(p-q\right)}^{2}}{4pq}. \end{array}$$

Throughout the paper, we refer to $A_{p}\left(f_{0},f_{1}\right)$ as the HP-integral. The HP-divergence measure belongs to the class of ϕ-divergences [36]. For the special case p=0.5, the divergence (2) becomes the symmetric $\chi^{2}$-divergence and is similar to the Rukhin *f*-divergence. See [37,38].

### 2.1. The Multivariate Runs Test Statistic

The MST is a graph of minimum weight among all graphs E that span *n* vertices. The MST has many applications including pattern recognition [39], clustering [40], nonparametric regression [41], and testing of randomness [42]. In this section, we focus on the FR multivariate two sample test statistic constructed from the MST.

Assume that sample realizations from $f_{0}$ and $f_{1}$, denoted by $X_{m}\in R^{m\times d}$ and $Y_{n}\in R^{n\times d}$, respectively, are available. Construct an MST spanning the samples from both $f_{0}$ and $f_{1}$ and color the edges in the MST that connect dichotomous samples green and color the remaining edges black. The FR test statistic $R_{m,n}:=R_{m,n}\left(X_{m},Y_{n}\right)$ is the number of green edges in the MST. Note that the test assumes a unique MST, therefore all inter point distances between data points must be distinct. We recall the following theorem from [7,8]:

**Theorem** **1.** *As m→∞ and n→∞ such that $\frac{m}{n+m}\to p$ and $\frac{n}{n+m}\to q$,*

(3) $$\begin{array}{c} 1-R_{m,n}\left(X_{m},Y_{n}\right)\;\frac{m+n}{2mn}\to D_{p}\left(f_{0},f_{1}\right),\;\;\;a.s. \end{array}$$

In the next section, we obtain bounds on the MSE convergence rates of the FR approximation for HP-divergence between densities that belong to $\Sigma_{d}\left(\eta ,K\right)$, the class of Hölder continuous functions with Lipschitz constant *K* and smoothness parameter 0<η≤1 [32]:

**Definition** **1** (Hölder class)**.** *Let $X\subset R^{d}$ be a compact space. The Hölder class $\Sigma_{d}\left(\eta ,K\right)$, with η-Hölder parameter, of functions with the $L_{d}$-norm, consists of the functions g that satisfy*

(4) $$\begin{array}{c} \begin{array}{c} \left\{g:∥g\left(z\right)-p_{x}^{\left\lfloor \eta \right\rfloor }\left(z\right){∥}_{d}\leq K\;∥x-z{∥}_{d}^{\eta },\;\;x,\;z\in X\right\}, \end{array} \end{array}$$

*where $p_{x}^{k}\left(z\right)$ is the Taylor polynomial (multinomial) of g of order k expanded about the point x and ⌊η⌋ is defined as the greatest integer strictly less than η.*

In what follows, we will use both notations $R_{m,n}$ and $R_{m,n}\left(X_{m},Y_{n}\right)$ for the FR statistic over the combined samples.

### 2.2. Convergence Rates

In this subsection, we obtain the mean convergence rate bounds for general non-uniform Lebesgue densities $f_{0}$ and $f_{1}$ belonging to the Hölder class $\Sigma_{d}\left(\eta ,K\right)$. Since the expectation of $R_{m,n}$ can be closely approximated by the sum of the expectation of the FR statistic constructed on a dense partition of ${\left[0,1\right]}^{d}$, $R_{m,n}$ is a quasi-additive functional in mean. The family of bounds (A16) in Appendix B enables us to achieve the minimax convergence rate for the mean under the Hölder class assumption with smoothness parameter 0<η≤1, d≥2:

**Theorem** **2** (Convergence Rate of the Mean)**.** *Let d≥2, and $R_{m,n}$ be the FR statistic for samples drawn from Hölder continuous and bounded density functions $f_{0}$ and $f_{1}$ in $\Sigma_{d}\left(\eta ,K\right)$. Then, for d≥2,*

(5) $$\begin{array}{c} \left|\frac{E\left[R_{m,n}\right]}{m+n}-2pq\int \frac{f_{0}\left(x\right)f_{1}\left(x\right)}{pf_{0}\left(x\right)+qf_{1}\left(x\right)}\;dx\right|\leq O\left({\left(m+n\right)}^{-\eta^{2}/\left(d\left(\eta +1\right)\right)}\right). \end{array}$$

This bound holds over the class of Lebesgue densities $f_{0},f_{1}\in \Sigma_{d}\left(\eta ,K\right)$, 0<η≤1. Note that this assumption can be relaxed to $f_{0}\in \Sigma_{d}^{s}\left(\eta ,K_{0}\right)$ and $f_{1}\in \Sigma_{d}^{s}\left(\eta ,K_{1}\right)$ that is Lebesgue densities $f_{0}$ and $f_{1}$ belong to the Strong Hölder class with the same Hölder parameter η and different constants $K_{0}$ and $K_{1}$, respectively.

The following variance bound uses the Efron–Stein inequality [43]. Note that in Theorem 3 we do not impose any strict assumptions. We only assume that the density functions are absolutely continuous and bounded with support on the unit cube ${\left[0,1\right]}^{d}$. Appendix C contains the proof.

**Theorem** **3.** *The variance of the HP-integral estimator based on the FR statistic, $R_{m,n}/\left(m+n\right)$ is bounded by*

(6) $$\begin{array}{c} Var\left(\frac{R_{m,n}\left(X_{m},Y_{n}\right)}{m+n}\right)\leq \frac{32\;c_{d}^{2}\;q}{\left(m+n\right)}, \end{array}$$

*where the constant $c_{d}$ depends only on d.*

**Figure 1:**
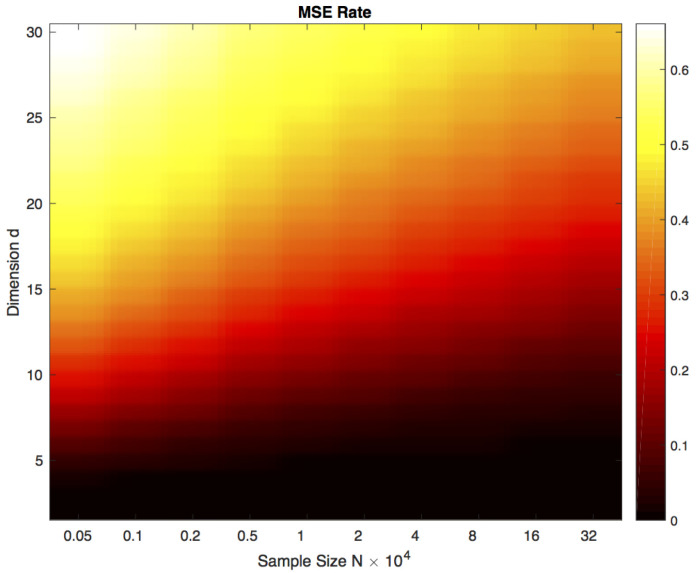
Heat map of the theoretical MSE rate of the FR estimator of the HP-divergence based on Theorems 2 and 3 as a function of dimension and sample size when N=m=n. Note the color transition (MSE) as sample size increases for high dimension. For fixed sample size *N*, the MSE rate degrades in higher dimensions.

By combining Theorems 2 and 3, we obtain the MSE rate of the form $O\left({m+n)}^{-\eta^{2}/\left(d\left(\eta +1\right)\right)}\right)+O\left({\left(m+n\right)}^{-1}\right)$. Figure 1 indicates a heat map showing the MSE rate as a function of *d* and N=m=n. The heat map shows that the MSE rate of the FR test statistic-based estimator given in (3) is small for large sample size *N*.

### 2.3. Proof Sketch of Theorem 2

In this subsection, we first establish subadditivity and superadditivity properties of the FR statistic, which will be employed to derive the MSE convergence rate bound. This will establish that the mean of the FR test statistic is a quasi-additive functional:

**Theorem** **4.** *Let $R_{m,n}\left(X_{m},Y_{n}\right)$ be the number of edges that link nodes from differently labeled samples $X_{m}=\left\{X_{1},\cdots ,X_{m}\right\}$ and $Y_{n}=\left\{Y_{1},\cdots ,Y_{n}\right\}$ in ${\left[0,1\right]}^{d}$. Partition ${\left[0,1\right]}^{d}$ into $l^{d}$ equal volume subcubes $Q_{i}$ such that $m_{i}$ and $n_{i}$ are the number of samples from $\left\{X_{1},\cdots ,X_{m}\right\}$ and $\left\{Y_{1},\cdots ,Y_{n}\right\}$, respectively, that fall into the partition $Q_{i}$. Then, there exists a constant $c_{1}$ such that*

(7) $$\begin{array}{c} E\left[R_{m,n}\left(X_{m},Y_{n}\right)\right]\leq \sum_{i=1}^{l^{d}}E\left[R_{m_{i},n_{i}}\left(\left(X_{m},Y_{n}\right)\cap Q_{i}\right)\right]+2\;c_{1}\;l^{d-1}\;{\left(m+n\right)}^{1/d}. \end{array}$$

*Here, $R_{m_{i},n_{i}}$ is the number of dichotomous edges in partition $Q_{i}$. Conversely, for the same conditions as above on partitions $Q_{i}$, there exists a constant $c_{2}$ such that*

(8) $$\begin{array}{c} E\left[R_{m,n}\left(X_{m},Y_{n}\right)\right]\geq \sum_{i=1}^{l^{d}}E\left[R_{m_{i},n_{i}}\left(\left(X_{m},Y_{n}\right)\cap Q_{i}\right)\right]-2\;c_{2}\;l^{d-1}\;{\left(m+n\right)}^{1/d}. \end{array}$$

The inequalities (7) and (8) are inspired by corresponding inequalities in [30,31]. The full proof is given in Appendix A. The key result in the proof is the inequality:

$$R_{m,n}\left(X_{m},Y_{n}\right)\leq \sum_{i=1}^{l^{d}}R_{m_{i},n_{i}}\left(\left(X_{m},Y_{n}\right)\cap Q_{i}\right)+2\left|D\right|,$$

where |D| indicates the number of all edges of the MST which intersect two different partitions.

**Figure 2:**
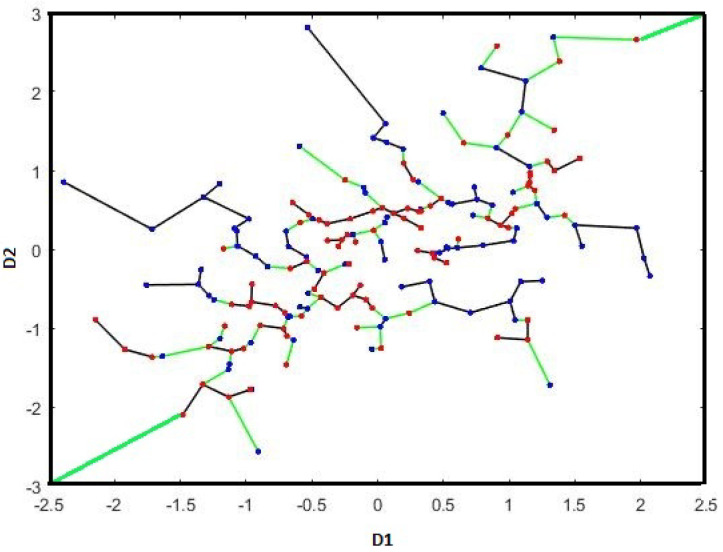
The dual MST spanning the merged set $X_{m}$ (blue points) and $Y_{n}$ (red points) drawn from two Gaussian distributions. The dual FR statistic ($R_{m,n}^{*}$) is the number of edges in the ${\mathrm{MST}}^{*}$ (contains nodes in $X_{m}\cup Y_{n}\cup \left\{2\;\mathrm{corner}\;\mathrm{points}\right\}$) that connect samples from different color nodes and corners (denoted in green). Black edges are the non-dichotomous edges in the ${\mathrm{MST}}^{*}$.

Furthermore, we adapt the theory developed in [17,30] to derive the MSE convergence rate of the FR statistic-based estimator by defining a dual MST and dual FR statistic, denoted by ${\mathrm{MST}}^{*}$ and $R_{m,n}^{*}$ respectively (see Figure 2):

**Definition** **2** (Dual MST, MST${}^{*}$ and dual FR statistic $R_{m,n}^{*}$)**.** Let $F_{i}$ be the set of corner points of the subsection $Q_{i}$ for $1\leq i\leq l^{d}$. Then, we define ${\mathrm{MST}}^{*}\left(X_{m}\cup Y_{n}\cap Q_{i}\right)$ as the boundary MST graph of partition $Q_{i}$ [17], which contains $X_{m}$ and $Y_{n}$ points falling inside the section $Q_{i}$ and those corner points in $F_{i}$ which minimize total MST length. Notice it is allowed to connect the MSTs in $Q_{i}$ and $Q_{j}$ through points strictly contained in $Q_{i}$ and $Q_{j}$ and corner points are taken into account under condition of minimizing total MST length. Another word, the dual MST can connect the points in $Q_{i}\cup Q_{j}$ by direct edges to pair to another point in $Q_{i}\cup Q_{j}$ or the corner the corner points (we assume that all corner points are connected) in order to minimize the total length. To clarify this, assume that there are two points in $Q_{i}\cup Q_{j}$, then the dual MST consists of the two edges connecting these points to the corner if they are closed to a corner point; otherwise, dual MST consists of an edge connecting one to another. Furthermore, we define $R_{m,n}^{*}\left(X_{m},Y_{n}\cap Q_{i}\right)$ as the number of edges in an ${\mathrm{MST}}^{*}$ graph connecting nodes from different samples and number of edges connecting to the corner points. Note that the edges connected to the corner nodes (regardless of the type of points) are always counted in dual FR test statistic $R_{m,n}^{*}$.

In Appendix B, we show that the dual FR test statistic is a quasi-additive functional in mean and $R_{m,n}^{*}\left(X_{m},Y_{n}\right)\geq R_{m,n}\left(X_{m},Y_{n}\right)$. This property holds true since $\mathrm{MST}(X_{m},Y_{n})$ and ${\mathrm{MST}}^{*}\left(X_{m},Y_{n}\right)$ graphs can only be different in the edges connected to the corner nodes, and in $R^{*}\left(X_{m},Y_{n}\right)$ we take all of the edges between these nodes and corner nodes into account.

To prove Theorem 2, we partition ${\left[0,1\right]}^{d}$ into $l^{d}$ subcubes. Then, by applying Theorem 4 and the dual MST, we derive the bias rate in terms of partition parameter *l* (see (A16) in Theorem A1). See Appendix B and Appendix E for details. According to (A16), for d≥2, and l=1,2,⋯, the slowest rates as a function of *l* are $l^{d}{\left(m+n\right)}^{\eta /d}$ and $l^{-\eta d}$. Therefore, we obtain an *l*-independent bound by letting *l* be a function of m+n that minimizes the maximum of these rates i.e.,

$$l\left(m+n\right)=arg\;\min_{l}\;\max\left\{l^{d}{\left(m+n\right)}^{-\eta /d},l^{-\eta d}\right\}.$$

The full proof of the bound in (2) is given in Appendix B.

### 2.4. Concentration Bounds

Another main contribution of our work in this part is to provide an exponential inequality convergence bound derived for the FR estimator of the HP-divergence. The error of this estimator can be decomposed into a bias term and a variance-like term via the triangle inequality:

$$\begin{array}{c} \begin{array}{c} \left|R_{m,n}-\int \frac{f_{0}\left(x\right)f_{1}\left(x\right)}{pf_{0}\left(x\right)+qf_{1}\left(x\right)}dx\right|\leq \underset{\mathrm{variance}-\mathrm{like}\;\mathrm{term}}{\underset{︸}{\left|R_{m,n}-E\left[R_{m,n}\right]\right|}}\\ \;\;+\underset{\mathrm{bias}\;\mathrm{term}}{\underset{︸}{\left|E\left[R_{m,n}\right]-\int \frac{f_{0}\left(x\right)f_{1}\left(x\right)}{pf_{0}\left(x\right)+qf_{1}\left(x\right)}dx\right|}}. \end{array} \end{array}$$

The bias bound was given in Theorem 2. Therefore, we focus on an exponential concentration bound for the variance-like term. One application of concentration bounds is to employ these bounds to compare confidence intervals on the HP-divergence measure in terms of the FR estimator. In [44,45], the authors provided an exponential inequality convergence bound for an estimator of Rény divergence for a smooth Hölder class of densities on the *d*-dimensional unite cube ${\left[0,1\right]}^{d}$. We show that if $X_{m}$ and $Y_{n}$ are the set of *m* and *n* points drawn from any two distributions $f_{0}$ and $f_{1}$, respectively, the FR criteria $R_{m,n}$ is tightly concentrated. Namely, we establish that, with high probability, $R_{m,n}$ is within

$$1-\;O\left({\left(m+n\right)}^{-2/d}{\epsilon^{*}}^{2}\right)$$

of its expected value, where $\epsilon^{*}$ is the solution of the following convex optimization problem:

(9) $$\begin{array}{cccc} \; & \min_{\epsilon \geq 0}\; & \; & C_{m,n}^{\prime }\left(\epsilon \right)\;\exp\left(\frac{-{\left(t/\left(2\epsilon \right)\right)}^{d/(d-1)}}{\left(m+n\right)\tilde{C}}\right)\\ \; & \mathrm{subject}\;\mathrm{to}\; & \; & \epsilon \geq O\left(7^{d+1}{\left(m+n\right)}^{1/d}\right), \end{array}$$

where $\tilde{C}=8{\left(4\right)}^{d/(d-1)}$ and

(10) $$\begin{array}{c} C_{m,n}^{\prime }\left(\epsilon \right)=8{\left(1-\;O\left({\left(m+n\right)}^{-2/d}\epsilon^{2}\right)\right)}^{-2}. \end{array}$$

Note that, under the assumption ${\left(m+n\right)}^{1/d}≃1$, $C_{m,n}^{\prime }\left(\epsilon \right)$ becomes a constant depending only on ϵ by $8{\left(1-\;(c\;\epsilon^{2}\right)}^{-2}$, where *c* is a constant. This is inferred from Theorems 5 and 6 below as ${\left(m+n\right)}^{1/d}≃1$. See Appendix D, specifically Lemmas A8–A12 for more detail. Indeed, we first show the concentration around the median. A median is by definition any real number $M_{e}$ that satisfies the inequalities $P(X\leq M_{e})\geq 1/2$ and $P(X\geq M_{e})\geq 1/2$. To derive the concentration results, the properties of growth bounds and smoothness for $R_{m,n}$, given in Appendix D, are exploited.

**Theorem** **5** (Concentration around the median)**.** Let $M_{e}$ be a median of $R_{m,n}$ which implies that $P\left(R_{m,n}\leq M_{e}\right)\geq 1/2$. Recall $\epsilon^{*}$ from (9) then we have

(11) $$\begin{array}{c} \begin{array}{c} P\left(|R_{m,n}\left(X_{m},Y_{n}\right)-M_{e}|\geq t\right)\leq C_{m,n}^{\prime }\left(\epsilon^{*}\right)\;\exp\left(\frac{-{\left(t/\epsilon^{*}\right)}^{d/(d-1)}}{\left(m+n\right)\tilde{C}}\right), \end{array} \end{array}$$

*where $\tilde{C}=8{\left(4\right)}^{d/(d-1)}$.*

**Theorem** **6** (Concentration of $R_{m,n}$ around the mean)**.** *Let $R_{m,n}$ be the FR statistic. Then,*

(12) $$\begin{array}{c} P\left(|R_{m,n}-E\left[R_{m,n}\right]|\geq t\right)\leq C_{m,n}^{\prime }\left(\epsilon^{*}\right)\exp\left(\frac{-{\left(t/\left(2\epsilon^{*}\right)\right)}^{d/(d-1)}}{\left(m+n\right)\;\tilde{C}}\right). \end{array}$$

Here, $\tilde{C}=8{\left(4\right)}^{d/(d-1)}$ and the explicit form for $C_{m,n}^{\prime }\left(\epsilon^{*}\right)$ is given by (10) when $\epsilon =\epsilon^{*}$.

See Appendix D for full proofs of Theorems 5 and 6. Here, we sketch the proofs. The proof of the concentration inequality for $R_{m,n}$, Theorem 6, requires involving the median $M_{e}$, where $P(R_{m,n}\leq M_{e})\geq 1/2$, inside the probability term by using

$$|R_{m,n}-E\left[R_{m,n}\right]|\leq \left|R_{m,n}-M_{e}|+\right|E\left[R_{m,n}\right]-M_{e}|.$$

To prove the expressions for the concentration around the median, Theorem 5, we first consider the $h^{d}$ uniform partitions of ${\left[0,1\right]}^{d}$, with edges parallel to the coordinate axes having edge lengths $h^{-1}$ and volumes $h^{-d}$. Then, by applying the Markov inequality, we show that with at least probability $1-\left(\delta_{m,n}^{h}/\epsilon \right)$, where $\delta_{m,n}^{h}=O\left(h^{d-1}{\left(m+n\right)}^{1/d}\right)$, the FR statistic $R_{m,n}$ is subadditive with 2ϵ threshold. Afterward, owing to the induction method [17], the growth bound can be derived with at least probability $1-\left(h\;\delta_{m,n}^{h}/\epsilon \right)$. The growth bound explains that with high probability there exists a constant depending on ϵ and *h*, $C_{\epsilon ,h}$, such that $R_{m,n}\leq C_{\epsilon ,h}{\left(m\;n\right)}^{1-1/d}$. Applying the law of total probability and semi-isoperimetric inequality (A108) in Lemma A11 gives us (A35). By considering the solution to convex optimization problem (9), i.e., $\epsilon^{*}$ and optimal h=7 the claimed results (11) and (12) are derived. The only constraint here is that ϵ is lower bounded by a function of $\delta_{m,n}^{h}=O\left(h^{d-1}{\left(m+n\right)}^{1/d}\right)$.

Next, we provide a bound for the variance-like term with high probability at least 1−δ. According to the previous results, we expect that this bound depends on $\epsilon^{*}$, *d*, *m* and *n*. The proof is short and is given in Appendix D.

**Theorem** **7** (Variance-like bound for $R_{m,n}$)**.** *Let $R_{m,n}$ be the FR statistic. With at least probability 1−δ, we have*

(13) $$\begin{array}{c} \left|R_{m,n}-E\left[R_{m,n}\right]\right|\leq O\left(\epsilon^{*}\;{\left(m+n\right)}^{(d-1)/d}{\left(\log\left(C_{m,n}^{\prime }\left(\epsilon^{*}\right)/\delta \right)\right)}^{(d-1)/d}\right). \end{array}$$

*or, equivalently,*

(14) $$\begin{array}{c} \left|\frac{R_{m,n}}{m+n}-\frac{E[R_{m,n}]}{m+n}\right|\leq O\left(\epsilon^{*}\;{\left(m+n\right)}^{-1/d}{\left(\log\left(C_{m,n}^{\prime }\left(\epsilon^{*}\right)/\delta \right)\right)}^{(d-1)/d}\right), \end{array}$$

where $C_{m,n}^{\prime }\left(\epsilon^{*}\right)$ depends on m,n, and d is given in (10) when $\epsilon =\epsilon^{*}$.

## 3. Numerical Experiments

### 3.1. Simulation Study

In this section, we apply the FR statistic estimate of the HP-divergence to both simulated and real data sets. We present results of a simulation study that evaluates the proposed bound on the MSE. We numerically validate the theory stated in Section 2.2 and Section 2.4 using multiple simulations. In the first set of simulations, we consider two multivariate Normal random vectors X, Y and perform three experiments d=2,4,8, to analyze the FR test statistic-based estimator performance as the sample sizes *m*, *n* increase. For the three dimensions d=2,4,8, we generate samples from two normal distributions with identity covariance and shifted means: $\mu_{1}=\left[0,0\right]$, $\mu_{2}=\left[1,0\right]$ and $\mu_{1}=\left[0,0,0,0\right]$, $\mu_{2}=\left[1,0,0,0\right]$ and $\mu_{1}=\left[0,0,\ldots ,0\right]$, $\mu_{2}=\left[1,0,\ldots ,0\right]$ when d=2, d=4 and d=8, respectively. For all of the following experiments, the sample sizes for each class are equal (m=n).

**Figure 3:**
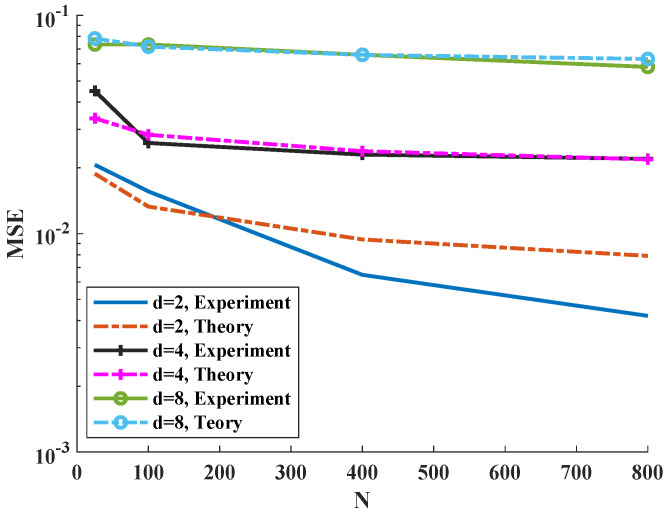
Comparison of the bound on the MSE theory and experiments for d=2,4,8 standard Gaussian random vectors versus sample size from 100 trials.

We vary N=m=n up to 800. From Figure 3, we deduce that, when the sample size increases, the MSE decreases such that for higher dimensions the rate is slower. Furthermore, we compare the experiments with the theory in Figure 3. Our theory generally matches the experimental results. However, the MSE for the experiments tends to decrease to zero faster than the theoretical bound. Since the Gaussian distribution has a smooth density, this suggests that a tighter bound on the MSE may be possible by imposing stricter assumptions on the density smoothness as in [12].

**Figure 4:**
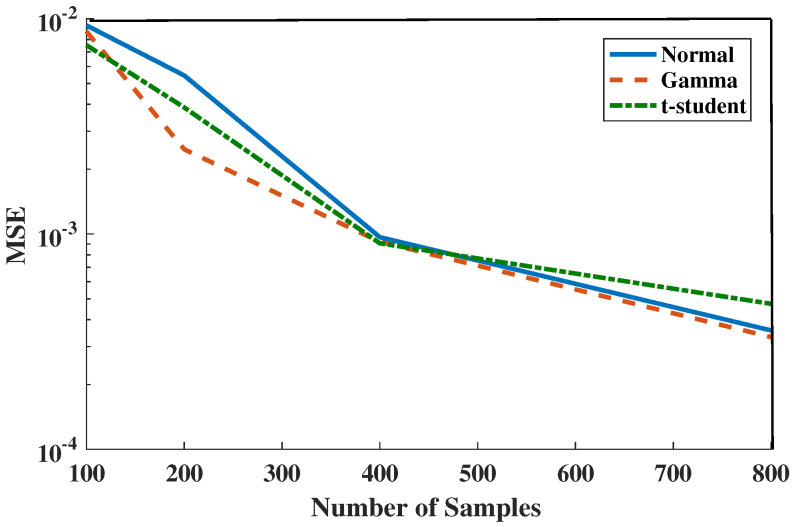
Comparison of experimentally predicted MSE of the FR-statistic as a function of sample size m=n in various distributions Standard Normal, Gamma ($\alpha_{1}=\alpha_{2}=1,\;\beta_{1}=\beta_{2}=1,\;\rho =0.5$) and Standard *t*-Student.

In our next simulation, we compare three bivariate cases: first, we generate samples from a standard Normal distribution. Second, we consider a distinct smooth class of distributions i.e., binomial Gamma density with standard parameters and dependency coefficient ρ=0.5. Third, we generate samples from Standard *t*-student distributions. Our goal in this experiment is to compare the MSE of the HP-divergence estimator between two identical distributions, $f_{0}=f_{1}$, when $f_{0}$ is one of the Gamma, Normal, and *t*-student density function. In Figure 4, we observe that the MSE decreases as *N* increases for all three distributions.

### 3.2. Real Datasets

We now show the results of applying the FR test statistic to estimate the HP-divergence using three different real datasets [46]:

- Human Activity Recognition (HAR), Wearable Computing, Classification of Body Postures and Movements (PUC-Rio): This dataset contains five classes (sitting-down, standing-up, standing, walking, and sitting) collected on eight hours of activities of four healthy subjects.
- Skin Segmentation dataset (SKIN): The skin dataset is collected by randomly sampling B,G,R values from face images of various age groups (young, middle, and old), race groups (white, black, and asian), and genders obtained from the FERET and PAL databases [47].
- Sensorless Drive Diagnosis (ENGIN) dataset: In this dataset, features are extracted from electric current drive signals. The drive has intact and defective components. The dataset contains 11 different classes with different conditions. Each condition has been measured several times under 12 different operating conditions, e.g., different speeds, load moments, and load forces.

We focus on two classes from each of the HAR, SKIN, and ENGIN datasets, specifically, for HAR dataset two classes “sitting” and “standing” and for SKIN dataset the classes “Skin” and “Non-skin” are considered. In the ENGIN dataset, the drive has intact and defective components, which results in 11 different classes with different conditions. We choose conditions 1 and 2.

**Figure 5:**
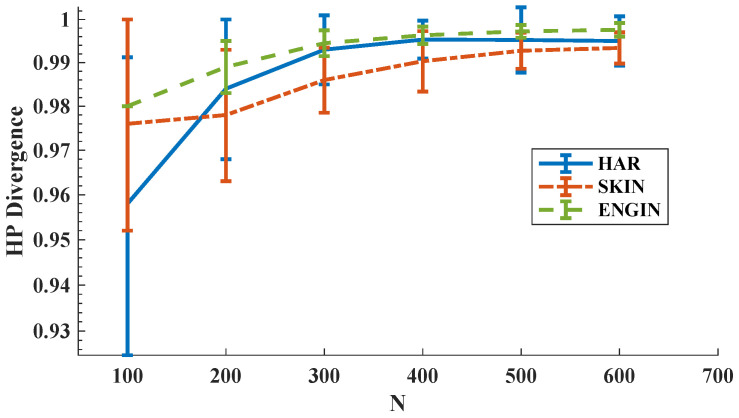
HP-divergence vs. sample size for three real datasets HAR, SKIN, and ENGIN.

**Figure 6:**
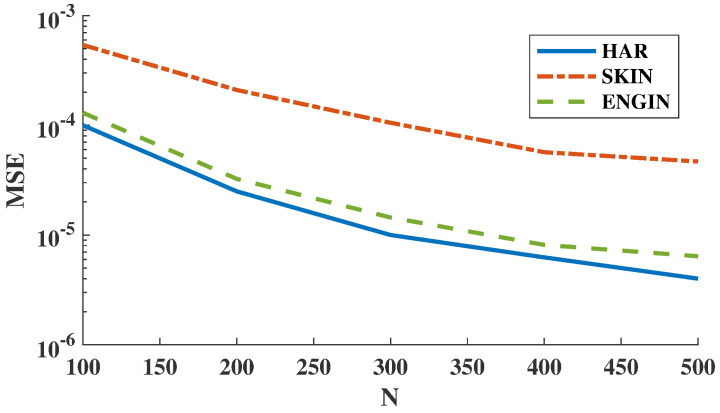
The empirical MSE vs. sample size. The empirical MSE of the FR estimator for all three datasets HAR, SKIN, and ENGIN decreases for larger sample size *N*.

In the first experiment, we computed the HP-divergence using KDE plug-in estimator and then the MSE for the FR test statistic estimator is derived as the sample size N=m=n increases. We used 95% confidence interval as the error bars. We observe in Figure 5 that the estimated HP-divergence ranges in [0,1], which is one of the HP-divergence properties [8]. Interestingly, when *N* increases the HP-divergence tends to 1 for all HAR, SKIN, and ENGIN datasets. Note that in this set of experiments we have repeated the experiments on independent parts of the datasets to obtain the error bars. Figure 6 shows that the MSE expectedly decreases as the sample size grows for all three datasets. Here, we have used the KDE plug-in estimator [12], implemented on the all available samples, to determine the true HP-divergence. Furthermore, according to Figure 6, the FR test statistic-based estimator suggests that the Bayes error rate is larger for the SKIN dataset compared to the HAR and ENGIN datasets.

**Figure 7:**
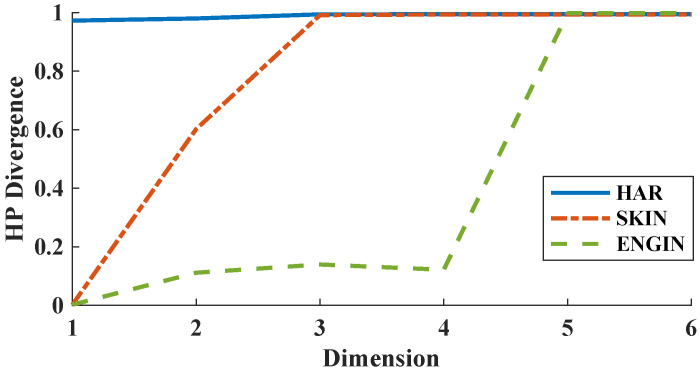
HP-divergence vs. dimension for three datasets HAR, SKIN, and ENGIN.

In our next experiment, we add the first six features (dimensions) in order to our datasets and evaluate the FR test statistic’s performance as the HP-divergence estimator. Surprisingly, the estimated HP-divergence doesn’t change for the HAR sample; however, big changes are observed for the SKIN and ENGIN samples (see Figure 7).

Finally, we apply the concentration bounds on the FR test statistic (i.e., Theorems 6 and 7) and compute theoretical implicit variance-like bound for the FR criteria with δ=0.05 error for the real datasets ENGIN, HAR, and SKIN. Since datasets ENGIN, HAR, and SKIN have the equal total sample size N=m+n=1200 and different dimensions d=14,12,4, respectively; here, we first intend to compare the concentration bound (13) on the FR statistic in terms of dimension *d* when δ=0.05. For real datasets ENGIN, HAR, and SKIN, we obtain

$$P\left(|R_{m,n}-E\left[R_{m,n}\right]|\leq \xi \right)\geq 0.95,$$

where $\xi =\xi^{\prime }\left[0.257,0.005,0.6\times 10^{-11}\right]$, respectively, and $\xi^{\prime }$ is a constant not dependent on *d*. One observes that as the dimension decreases the interval becomes significantly tighter. However, this could not be generally correct and computing bound (13) precisely requires the knowledge of distributions and unknown constants. In Table 1, we compute the standard variance-like bound by applying the percentiles technique and observe that the bound threshold is not monotonic in terms of dimension *d*. Table 1 shows the FR test statistic, HP-divergence estimate (denoted by $R_{m,n}$, ${\hat{D}}_{p}$, respectively), and standard variance-like interval for the FR statistic using the three real datasets HAR, SKIN, and ENGIN.

## 4. Conclusions

We derived a bound on the MSE convergence rate for the Friedman–Rafsky estimator of the Henze–Penrose divergence assuming the densities are sufficiently smooth. We employed a partitioning strategy to derive the bias rate which depends on the number of partitions, the sample size m+n, the Hölder smoothness parameter η, and the dimension *d*. However, by using the optimal partition number, we derived the MSE convergence rate only in terms of m+n, η, and *d*. We validated our proposed MSE convergence rate using simulations and illustrated the approach for the meta-learning problem of estimating the HP-divergence for three real-world data sets. We also provided concentration bounds around the median and mean of the estimator. These bounds explicitly provide the rate that the FR statistic approaches its median/mean with high probability, not only as a function of the number of samples, *m*, *n*, but also in terms of the dimension of the space *d*. By using these results, we explored the asymptotic behavior of a variance-like rate in terms of *m*, *n*, and *d*.

## Figures and Tables

**Table 1:** $R_{m,n}$, ${\hat{D}}_{p}$, *m*, and *n* are the FR test statistic, HP-divergence estimates using $R_{m,n}$, and sample sizes for two classes, respectively.

FR Test Statistic					
**Dataset**	$E[R_{m,n}]$	${\hat{D}}_{p}$	m	n	**Variance-Like Interval**
HAR	3	0.995	600	600	(2.994,3.006)
SKIN	4.2	0.993	600	600	(4.196,4.204)
ENGIN	1.8	0.997	600	600	(1.798,1.802)

**Figure A1:**
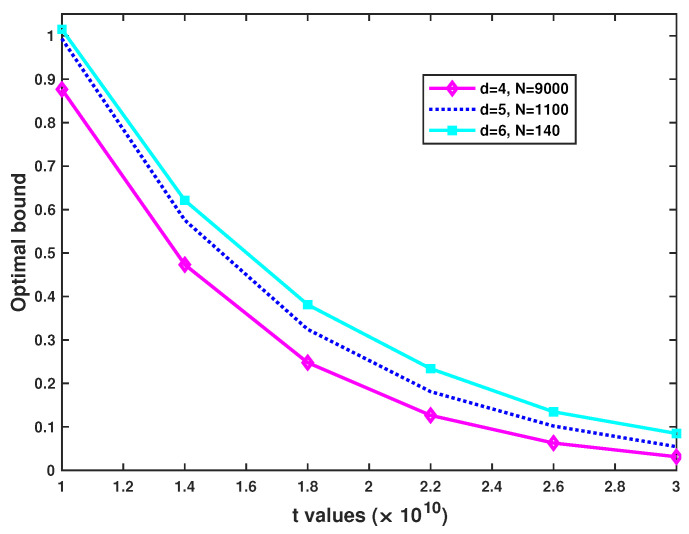
Optimal bound for (12), when h=7 versus $t\in 10^{10}\left[1,3\right]$. The bound decreases as *t* grows.

## Abbreviations

HP	Henze-Penrose
BER	Bayes error rate
MST	Minimal Spanning Tree
FR	Friedman-Rafsky
MSE	Mean squared error

## Appendix A. Proof of Theorem 4

In this section, we prove the subadditivity and superadditivity for the mean of FR test statistic. For this, first we need to illustrate the following lemma.

**Lemma** **A1.**
*Let ${\left\{Q_{i}\right\}}_{i=1}^{l^{d}}$ be a uniform partition of ${\left[0,1\right]}^{d}$ into $l^{d}$ subcubes $Q_{i}$ with edges parallel to the coordinate axes having edge lengths $l^{-1}$ and volumes $l^{-d}$. Let $D_{ij}$ be the set of edges of MST graph between $Q_{i}$ and $Q_{j}$ with cardinality $|D_{ij}|$, then for |D| defined as the sum of $|D_{ij}|$ for all $i,j=1,\cdots ,l^{d}$, i≠j, we have $E|D|=O(l^{d-1}\;n^{1/d})$, or more explicitly*

(A1) $$\begin{array}{c} E\left[|D|\right]\leq C^{\prime }l^{d-1}n^{1/d}+O\left(l^{d-1}n^{(1/d)-s}\right), \end{array}$$

*where η>0 is the Hölder smoothness parameter and*

$$s=\frac{(1-1/d)\eta }{d\;((1-1/d)\eta +1)}.$$

Here, and in what follows, denote $\Xi_{MST}\left(X_{n}\right)$ the length of the shortest spanning tree on $X_{n}=\left\{X_{1},\cdots ,X_{n}\right\}$, namely

$$\begin{array}{c} \Xi_{MST}\left(X_{n}\right):=\min_{T}\sum_{e\in T}\left|e\right|, \end{array}$$

where the minimum is over all spanning trees *T* of the vertex set $X_{n}$. Using the subadditivity relation for $\Xi_{MST}$ in [17], with the uniform partition of ${\left[0,1\right]}^{d}$ into $l^{d}$ subcubes $Q_{i}$ with edges parallel to the coordinate axes having edge lengths $l^{-1}$ and volumes $l^{-d}$, we have

(A2) $$\begin{array}{c} \Xi_{MST}\left(X_{n}\right)\leq \sum_{i=1}^{l^{d}}\Xi_{MST}\left(X_{n}\cap Q_{i}\right)+C\;l^{d-1}, \end{array}$$

where *C* is constant. Denote *D* the set of all edges of $MST\left(\overset{M}{\underset{i=1}{⋃}}Q_{i}\right)$ that intersect two different subcubes $Q_{i}$ and $Q_{j}$ with cardinality |D|. Let $|e_{i}|$ be the length of *i*-th edge in set *D*. We can write

$$\begin{array}{c} \sum_{i\in |D|}|e_{i}|\leq Cl^{d-1}\;\;\mathrm{and}\;\;E\sum_{i\in |D|}\left|e_{i}\right|\leq Cl^{d-1}, \end{array}$$

also we know that

(A3) $$\begin{array}{c} E\sum_{i\in |D|}\left|e_{i}\right|=E_{D}\sum_{i\in |D|}E\left[|e_{i}||D\right]. \end{array}$$

Note that using the result from ([31], Proposition 3), for some constants $C_{i1}$ and $C_{i2}$, we have

(A4) $$\begin{array}{c} E|e_{i}|\leq C_{i1}n^{-1/d}+C_{i2}n^{-(1/d)-s},\;\;\;i\in \left|D\right|. \end{array}$$

Now, let $C_{1}=\max_{i}\left\{C_{i1}\right\}$ and $C_{2}=\max_{i}\left\{C_{i2}\right\}$, hence we can bound the expectation (A3) as

$$\begin{array}{c} E|D|\;\left(C_{1}n^{-1/d}+C_{2}\left(n^{-(1/d)-s}\right)\right)\leq Cl^{d-1}, \end{array}$$

which implies

$$\begin{array}{c} \begin{array}{c} E|D|\leq (C_{1}n^{-1/d}+O\left(n^{-(1/d)-s}\right))\\ \;\;\leq C^{\prime }l^{d-1}n^{1/d}+O\left(l^{d-1}n^{(1/d)-s}\right). \end{array} \end{array}$$

To aim toward the goal (7), we partition ${\left[0,1\right]}^{d}$ into $M:=l^{d}$ subcubes $Q_{i}$ of side 1/l. Recalling Lemma 2.1 in [48], we therefore have the set inclusion:

(A5) $$\begin{array}{c} MST\left(\overset{M}{\underset{i=1}{⋃}}Q_{i}\right)\subset \overset{M}{\underset{i=1}{⋃}}MST\left(Q_{i}\right)\cup D, \end{array}$$

where *D* is defined as in Lemma A1. Let $m_{i}$ and $n_{i}$ be the number of sample $\left\{X_{1},\cdots ,X_{m}\right\}$ and $\left\{Y_{1},\cdots ,Y_{n}\right\}$ respectively falling into the partition $Q_{i}$, such that $\sum_{i}m_{i}=m$ and $\sum_{i}n_{i}=n$. Introduce sets *A* and *B* as

$$A:=MST\left(\overset{M}{\underset{i=1}{⋃}}Q_{i}\right),\;\;\;B:=\overset{M}{\underset{i=1}{⋃}}MST\left(Q_{i}\right).$$

Since set *B* has fewer edges than set *A*, thus (A5) implies that the difference set of *B* and *A* contains at most 2|D| edges, where |D| is the number of edges in *D*. On the other word,

$$\begin{array}{c} \begin{array}{c} \left|A\Delta B|\leq |A-B|+|B-A|=|D|+|B-A\right|\\ =|D|+(|B|-|B\cap A|\leq |D|+(|A|-|B\cap A|)=2|D|. \end{array} \end{array}$$

The number of edge linked nodes from different samples in set *A* is bounded by the number of edge linked nodes from different samples in set *B* plus 2|D|:

(A6) $$\begin{array}{c} R_{m,n}\left(X_{m},Y_{n}\right)\leq \sum_{i=1}^{M}R_{m_{i},n_{i}}\left(\left(X_{m},Y_{n}\right)\cap Q_{i}\right)+2\left|D\right|. \end{array}$$

Here, $R_{m_{i},n_{i}}$ stands with the number edge linked nodes from different samples in partition $Q_{i}$, *M*. Next, we address the reader to Lemma A1, where it has been shown that there is a constant *c* such that $E|D|\leq c\;l^{d-1}\;{\left(m+n\right)}^{1/d}$. This concludes the claimed assertion (7). Now, to accomplish the proof, the lower bound term in (8) is obtained with similar methodology and the set inclusion:

(A7) $$\begin{array}{c} \overset{M}{\underset{i=1}{⋃}}MST\left(Q_{i}\right)\subset MST\left(\overset{M}{\underset{i=1}{⋃}}Q_{i}\right)\cup D. \end{array}$$

This completes the proof.

## Appendix B. Proof of Theorem 2

As many of continuous subadditive functionals on ${\left[0,1\right]}^{d}$, in the case of the FR statistic, there exists a dual superadditive functional $R_{m,n}^{*}$ based on dual MST, ${\mathrm{MST}}^{*}$, proposed in Definition 2. Note that, in the MST* graph, the degrees of the corner points are bounded by $c_{d}$, where it only depends on dimension *d*, and is the bound for degree of every node in MST graph. The following properties hold true for dual FR test statistic, $R_{m,n}^{*}$:

**Lemma** **A2.**
*Given samples $X_{m}=\left\{X_{1},\cdots ,X_{m}\right\}$ and $Y_{n}=\left\{Y_{1},\cdots ,Y_{n}\right\}$, the following inequalities hold true:*

(i) *For constant $c_{d}$ which depends on d:*
  (A8) $$\begin{array}{c} \begin{array}{c} R_{m,n}^{*}\left(X_{m},Y_{n}\right)\leq R_{m,n}\left(X_{m},Y_{n}\right)+c_{d}\;2^{d},\\ R_{m,n}\left(X_{m},Y_{n}\right)\leq R_{m,n}^{*}\left(X_{m},Y_{n}\right). \end{array} \end{array}$$
(ii) *(Subadditivity on $E[R_{m,n}^{*}]$ and Superadditivity) Partition ${\left[0,1\right]}^{d}$ into $l^{d}$ subcubes $Q_{i}$ such that $m_{i}$, $n_{i}$ be the number of sample $X_{m}=\left\{X_{1},\cdots ,X_{m}\right\}$ and $Y_{n}=\left\{Y_{1},\cdots ,Y_{n}\right\}$ respectively falling into the partition $Q_{i}$ with dual $R_{m_{i},n_{i}}^{*}$. Then, we have*
  (A9) $$\begin{array}{c} E\left[R_{m,n}^{*}\left(X_{m},Y_{n}\right)\right]\leq \sum_{i=1}^{l^{d}}E\left[R_{m_{i},n_{i}}^{*}\left(\left(X_{m},Y_{n}\right)\cap Q_{i}\right)\right]+c\;l^{d-1}\;{\left(m+n\right)}^{1/d},\\ R_{m,n}^{*}\left(X_{m},Y_{n}\right)\geq \sum_{i=1}^{l^{d}}R_{m_{i},n_{i}}^{*}\left(\left(X_{m},Y_{n}\right)\cap Q_{i}\right)-2^{d}c_{d}l^{d}, \end{array}$$
  *where c is a constant.*

*(i) Consider the nodes connected to the corner points. Since $\mathrm{MST}(X_{m},Y_{n})$ and ${\mathrm{MST}}^{*}\left(X_{m},Y_{n}\right)$ can only be different in the edges connected to these nodes, and in $R^{*}\left(X_{m},Y_{n}\right)$ we take all of the edges between these nodes and corner nodes into account, so we obviously have the second relation in (A8). In addition, for the first inequality in (A8), it is enough to say that the total number of edges connected to the corner nodes is upper bounded by $2^{d}\;c_{d}$.*

*(ii) Let $|D^{*}|$ be the set of edges of the ${\mathrm{MST}}^{*}$ graph which intersect two different partitions. Since MST and ${\mathrm{MST}}^{*}$ are only different in edges of points connected to the corners and edges crossing different partitions. Therefore, $|D^{*}\left|\leq |D\right|$. By eliminating one edge in set D in the worse scenario we would face two possibilities: either the corresponding node is connected to the corner which is counted anyways or any other point in MST graph which wouldn’t change the FR test statistic. This implies the following subadditivity relation:*

$$\begin{array}{c} R_{m,n}^{*}\left(X_{m},Y_{n}\right)-\left|D\right|\leq \sum_{i=1}^{l^{d}}R_{m_{i},n_{i}}^{*}\left(\left(X_{m},Y_{n}\right)\cap Q_{i}\right). \end{array}$$

*Further from Lemma A1, we know that there is a constant c such that $E|D|\leq c\;l^{d-1}\;{\left(m+n\right)}^{1/d}$. Hence, the first inequality in (A9) is obtained. Next, consider $|D_{c}^{*}|$ which represents the total number of edges from both samples only connected to the all corners points in ${\mathrm{MST}}^{*}$ graph. Therefore, one can easily claim:*

$$\begin{array}{c} R_{m,n}^{*}\left(X_{m},Y_{n}\right)\geq \sum_{i=1}^{l^{d}}R_{m_{i},n_{i}}^{*}\left(\left(X_{m},Y_{n}\right)\cap Q_{i}\right)-\left|D_{c}^{*}\right|. \end{array}$$

*In addition, we know that $|D_{c}^{*}|\leq 2^{d}l^{d}c_{d}$ where $c_{d}$ stands with the largest possible degree of any vertex. One can write*

$$\begin{array}{c} R_{m,n}^{*}\left(X_{m},Y_{n}\right)\geq \sum_{i=1}^{l^{d}}R_{m_{i},n_{i}}^{*}\left(\left(X_{m},Y_{n}\right)\cap Q_{i}\right)-2^{d}c_{d}l^{d}. \end{array}$$

The following list of Lemmas A3, A4 and A6 are inspired from [49] and are required to prove Theorem A1. See Appendix E for their proofs.

**Lemma** **A3.**
*Let g(x) be a density function with support ${\left[0,1\right]}^{d}$ and belong to the Hölder class $\Sigma_{d}\left(\eta ,L\right)$, 0<η≤1, stated in Definition 1. In addition, assume that P(x) is a η-Hölder smooth function, such that its absolute value is bounded from above by a constant. Define the quantized density function with parameter l and constants $\phi_{i}$ as*

(A10) $$\begin{array}{c} \hat{g}\left(x\right)=\sum_{i=1}^{M}\phi_{i}1\left\{x\in Q_{i}\right\},\;\;\;where\;\phi_{i}=l^{d}\;\int_{Q_{i}}g\left(x\right)\;dx. \end{array}$$

*Let $M=l^{d}$ and $Q_{i}=\{x,x_{i}:∥x-x_{i}∥<l^{-d}\}$. Then,*

(A11) $$\begin{array}{c} \int ∥\left(g\left(x\right)-\hat{g}\left(x\right)\right)P\left(x\right)∥\;dx\leq O\left(l^{-d\eta }\right). \end{array}$$

**Lemma** **A4.**
*Denote Δ(x,S) the degree of vertex x∈S in the MST over set S with the n number of vertices. For given function P(x,x), one obtains*

(A12) $$\int P\left(x,x\right)g\left(x\right)E\left[\Delta \left(x,S\right)\right]\;dx=2\;\int P\left(x,x\right)g\left(x\right)\;dx+\varsigma_{\eta }\left(l,n\right),$$

*where, for constant η>0,*

(A13) $$\begin{array}{c} \varsigma_{\eta }\left(l,n\right)=\left(O\left(l/n\right)-2\;l^{d}/n\right)\int g\left(x\right)P\left(x,x\right)\;dx+O\left(l^{-d\eta }\right). \end{array}$$

**Lemma** **A5.**
*Assume that, for given k, $g_{k}\left(x\right)$ is a bounded function belong to $\Sigma_{d}\left(\eta ,L\right)$. Let $P:R^{d}\times R^{d}\mapsto \left[0,1\right]$ be a symmetric, smooth, jointly measurable function, such that, given k, for almost every $x\in R^{d}$, P(x,.) is measurable with x a Lebesgue point of the function $g_{k}\left(.\right)P\left(x,.\right)$. Assume that the first derivative P is bounded. For each k, let $Z_{1}^{k},Z_{2}^{k},\cdots ,Z_{k}^{k}$ be an independent d-dimensional variable with common density function $g_{k}$. Set $Z_{k}=\left\{Z_{1}^{k},Z_{2}^{k}\cdots ,Z_{k}^{k}\right\}$ and $Z_{k}^{x}=\left\{x,Z_{2}^{k},Z_{3}^{k}\cdots ,Z_{k}^{k}\right\}$. Then,*

(A14) $$\begin{array}{c} E\left[\sum_{j=2}^{k}P\left(x,Z_{j}^{k}\right)1\left\{\left(x,Z_{j}^{k}\right)\in MST\left(Z_{k}^{x}\right)\right\}\right]=P\left(x,x\right)\;E\left[\Delta (x,Z_{k}^{x})\right]+\left\{O\left(k^{-\eta /d}\right)+O\left(k^{-1/d}\right)\right\}. \end{array}$$

**Lemma** **A6.**
*Consider the notations and assumptions in Lemma A5. Then,*

(A15) $$\begin{array}{c} \left|k^{-1}\underset{\;1\leq i<j\leq k}{\sum \sum }P\left(Z_{i}^{k},Z_{j}^{k}\right)1\left\{\left(Z_{i}^{k},Z_{j}^{k}\right)\in MST\left(Z_{k}\right)\right\}-\int_{R^{d}}P\left(x,x\right)g_{k}\left(x\right)\;dx\right|\\ \;\;\;\leq \varsigma_{\eta }\left(l,k\right)+O\left(k^{-\eta /d}\right)+O\left(k^{-1/d}\right). \end{array}$$

*Here, MST(S) denotes the MST graph over nice and finite set $S\subset R^{d}$ and η is the smoothness Hölder parameter. Note that $\varsigma_{\eta }\left(l,k\right)$ is given as before in Lemma A4 (A13).*

**Theorem** **A1.**
*Assume $R_{m,n}:=R\left(X_{m},Y_{n}\right)$ denotes the FR test statistic and densities $f_{0}$ and $f_{1}$ belong to the Hölder class $\Sigma_{d}\left(\eta ,L\right)$, 0<η≤1. Then, the rate for the bias of the $R_{m,n}$ estimator for d≥2 is of the form:*

(A16) $$\begin{array}{c} \left|\frac{E\left[R_{m,n}\right]}{m+n}-2pq\int \frac{f_{0}\left(x\right)f_{1}\left(x\right)}{pf_{0}\left(x\right)+qf_{1}\left(x\right)}\;dx\right|\leq O\left(l^{d}{\left(m+n\right)}^{-\eta /d}\right)+O\left(l^{-d\eta }\right). \end{array}$$

*The proof and a more explicit form for the bound (A16) are given in Appendix E.*

Now, we are at the position to prove the assertion in (5). Without loss of generality, assume that $\left(m+n\right)l^{-d}>1$. In the range d≥2 and 0<η≤1, we select *l* as a function of m+n to be the sequence increasing in m+n which minimizes the maximum of these rates:

$$\begin{array}{c} l\left(m+n\right)=arg\;\min_{l}\max\left\{l^{d}{\left(m+n\right)}^{-\eta /d},\;l^{-\eta d}\right\}. \end{array}$$

The solution l=l(m+n) occurs when $l^{d}{\left(m+n\right)}^{-\eta /d}=l^{-\eta d}$, or equivalently $l=\lfloor {\left(m+n\right)}^{\eta /(d^{2}\left(\eta +1\right))}\rfloor$. Substitute this into *l* in the bound (A16), the RHS expression in (5) for d≥2 is established.

## Appendix C. Proof of Theorems 3

To bound the variance, we will apply one of the first concentration inequalities which was proved by Efron and Stein [43] and further was improved by Steele [18].

**Lemma** **A7** (The Efron–Stein Inequality)**.** *Let $X_{m}=\left\{X_{1},\cdots ,X_{m}\right\}$ be a random vector on the space S. Let $X^{\prime }=\left\{X_{1}^{\prime },\cdots ,X_{m}^{\prime }\right\}$ be the copy of random vector $X_{m}$. Then, if f:S×⋯×S→R, we have*

(A17) $$\begin{array}{c} V\left[f(X_{m})\right]\leq \frac{1}{2}\sum_{i=1}^{m}E\left[{\left(f\left(X_{1},\cdots ,X_{m}\right)-f\left(X_{1},\cdots ,X_{i}^{\prime },\cdots ,X_{m}\right)\right)}^{2}\right]. \end{array}$$

Consider two set of nodes $X_{i}$, 1≤i≤m and $Y_{j}$ for 1≤j≤n. Without loss of generality, assume that m<n. Then, consider the n−m virtual random points $X_{m+1},\ldots ,X_{n}$ with the same distribution as $X_{i}$, and define $Z_{i}:=\left(X_{i},Y_{i}\right)$. Now, for using the Efron–Stein inequality on set $Z_{n}=\left\{Z_{1},\ldots ,Z_{n}\right\}$, we involve another independent copy of $Z_{n}$ as $Z_{n}^{\prime }=\left\{Z_{1}^{\prime },\ldots ,Z_{n}^{\prime }\right\}$, and define $Z_{n}^{\left(i\right)}:=\left(Z_{1},\ldots ,Z_{i-1},Z_{i}^{\prime },Z_{i+1},\ldots ,Z_{n}\right)$, then $Z_{n}^{\left(1\right)}$ becomes $\left(Z_{1}^{\prime },Z_{2},\ldots ,Z_{n}\right)=\left\{\left(X_{1}^{\prime },Y_{1}^{\prime }\right),\left(X_{2},Y_{2}\right),\cdots ,\left(X_{m},Y_{n}\right)\right\}=:\left(X_{m}^{\left(1\right)},Y_{n}^{\left(1\right)}\right)$ where $\left(X_{1}^{\prime },Y_{1}^{\prime }\right)$ is independent copy of $\left(X_{1},Y_{1}\right)$. Next, define the function $r_{m,n}\left(Z_{n}\right):=R_{m,n}/\left(m+n\right)$, which means that we discard the random samples $X_{m+1},\ldots ,X_{n}$, and find the previously defined $R_{m,n}$ function on the nodes $X_{i}$, 1≤i≤m and $Y_{j}$ for 1≤j≤n, and multiply by some coefficient to normalize it. Then, according to the Efron–Stein inequality, we have

$$\begin{array}{c} Var\left(r_{m,n}\left(Z_{n}\right)\right)\leq \frac{1}{2}\sum_{i=1}^{n}E\left[{\left(r_{m,n}\left(Z_{n}\right)-r_{m,n}\left(Z_{n}^{\left(i\right)}\right)\right)}^{2}\right]. \end{array}$$

Now, we can divide the RHS as

(A18) $$\begin{array}{c} \frac{1}{2}\sum_{i=1}^{n}E\left[{\left(r_{m,n}\left(Z_{n}\right)-r_{m,n}\left(Z_{n}^{\left(i\right)}\right)\right)}^{2}\right]=\frac{1}{2}\sum_{i=1}^{m}E\left[{\left(r_{m,n}\left(Z_{n}\right)-r_{m,n}\left(Z_{n}^{\left(i\right)}\right)\right)}^{2}\right]\\ \;\;+\frac{1}{2}\sum_{i=m+1}^{n}E\left[{\left(r_{m,n}\left(Z_{n}\right)-r_{m,n}\left(Z_{n}^{\left(i\right)}\right)\right)}^{2}\right]. \end{array}$$

The first summand becomes

$$\begin{array}{c} \begin{array}{c} =\frac{1}{2}\sum_{i=1}^{m}E\left[{\left(r_{m,n}\left(Z_{n}\right)-r_{m,n}\left(Z_{n}^{\left(i\right)}\right)\right)}^{2}\right]=\frac{m}{2\;{\left(m+n\right)}^{2}}E\left[{\left(R_{m,n}\left(X_{m},Y_{n}\right)-R_{m,n}\left(X_{m}^{\left(1\right)},Y_{n}^{\left(1\right)}\right)\right)}^{2}\right], \end{array} \end{array}$$

which can also be upper bounded as follows:

(A19) $$\begin{array}{c} \begin{array}{c} \left|R_{m,n}\left(X_{m},Y_{n}\right)-R_{m,n}\left(X_{m}^{\left(1\right)},Y_{n}^{\left(1\right)}\right)\right|\leq \left|R_{m,n}\left(X_{m},Y_{n}\right)-R_{m,n}\left(X_{m}^{\left(1\right)},Y_{n}\right)\right|\\ \;\;\;+\left|R\left(X_{m}^{\left(1\right)},Y_{n}\right)-R_{m,n}\left(X_{m}^{\left(1\right)},Y_{n}^{\left(1\right)}\right)\right|. \end{array} \end{array}$$

For deriving an upper bound on the second line in (A19), we should observe how much changing a point’s position modifies the amount of $R_{m,n}\left(X_{m},Y_{n}\right)$. We consider two steps of changing $X_{1}$’s position: we first remove it from the graph, and then add it to the new position. Removing it would change $R_{m,n}\left(X_{m},Y_{n}\right)$ at most by $2\;c_{d}$ because $X_{1}$ has a degree of at most $c_{d}$, and $c_{d}$ edges will be removed from the MST graph, and $c_{d}$ edges will be added to it. Similarly, adding $X_{1}$ to the new position will affect $R_{m,n}\left(X_{m,n},Y_{m,n}\right)$ at most by $2c_{d}$. Thus, we have

$$\left|R_{m,n}\left(X_{m},Y_{n}\right)-R_{m,n}\left(X_{m}^{\left(1\right)},Y_{n}\right)\right|\leq 4\;c_{d},$$

and we can also similarly reason that

$$\left|R_{m,n}\left(X_{m}^{\left(1\right)},Y_{n}\right)-R_{m,n}\left(X_{m}^{\left(1\right)},Y_{n}^{\left(1\right)}\right)\right|\leq 4\;c_{d}.$$

Therefore, totally we would have

$$\begin{array}{c} \left|R_{m,n}\left(X_{m},Y_{n}\right)-R_{m,n}\left(X_{m}^{\left(1\right)},Y_{n}^{\left(1\right)}\right)\right|\leq 8\;c_{d}. \end{array}$$

Furthermore, the second summand in (A18) becomes

$$\begin{array}{c} \begin{array}{c} \;=\frac{1}{2}\sum_{i=m+1}^{n}E\left[{\left(r_{m,n}\left(Z_{n}\right)-r_{m,n}\left(Z_{n}^{\left(i\right)}\right)\right)}^{2}\right]=K_{m,n}E\left[{\left(R_{m,n}\left(X_{m},Y_{n}\right)-R_{m,n}\left(X_{m}^{\left(m+1\right)},Y_{n}^{\left(m+1\right)}\right)\right)}^{2}\right], \end{array} \end{array}$$

where $K_{m,n}=\frac{n-m}{2\;{\left(m+n\right)}^{2}}$. Since, in $\left(X_{m}^{\left(m+1\right)},Y_{n}^{\left(m+1\right)}\right)$, the point $X_{m+1}^{\prime }$ is a copy of virtual random point $X_{m+1}$, therefore this point doesn’t change the FR test statistic $R_{m,n}$. In addition, following the above arguments, we have

$$\begin{array}{c} \left|R_{m,n}\left(X_{m},Y_{n}\right)-R_{m,n}\left(X_{m},Y_{n}^{\left(m+1\right)}\right)\right|\leq 4\;c_{d}. \end{array}$$

Hence, we can bound the variance as below:

(A20) $$\begin{array}{c} Var\left(r_{m,n}\left(Z_{n}\right)\right)\leq \frac{8c_{d}^{2}\left(n-m\right)}{{\left(m+n\right)}^{2}}+\frac{32\;c_{d}^{2}\;m}{{\left(m+n\right)}^{2}}. \end{array}$$

Combining all results with the fact that $\frac{n}{m+n}\to q$ concludes the proof.

## Appendix D. Proof of Theorems 5–7

We will need the following prominent results for the proofs.

**Lemma** **A8.**
*For h=1,2,⋯, let $\delta_{m,n}^{h}$ be the function $c\;h^{d-1}{\left(m+n\right)}^{1/d}$, where c is a constant. Then, for ϵ>0, we have*

(A21) $$P\left(R_{m,n}\left(X_{m},Y_{n}\right)\leq \sum_{i=1}^{h^{d}}R_{m_{i},n_{i}}\left(X_{m_{i}},Y_{n_{i}}\right)+2\epsilon \right)\geq \frac{\epsilon -\delta_{m,n}^{h}}{\epsilon }.$$

*Note that, in the case $\epsilon \leq \delta_{m,n}^{h}$, the above claimed inequality becomes trivial.*

The subadditivity property for FR test statistic $R_{m,n}$ in Lemma A8, as well as Euclidean functionals, leads to several non-trivial consequences. The growth bound was first explored by Rhee (1993b) [50], and as is illustrated in [17,27] has a wide range of applications. In this paper, we investigate the probabilistic growth bound for $R_{m,n}$. This observation will lead us to our main goal in this appendix that is providing the proof of Theorem 6. For what follows, we will use $\delta_{m,n}^{h}$ notation for the expression $O\left(h^{d-1}{\left(m+n\right)}^{1/d}\right)$.

**Lemma** **A9** (Growth bounds for $R_{m,n}$)**.** *Let $R_{m,n}$ be the FR test statistic. Then, for given non-negative ϵ, such that $\epsilon \geq h^{2}\;\delta_{m,n}^{h}$, with at least probability $g\left(\epsilon \right):=1-\frac{h\;\delta_{m,n}^{h}}{\epsilon }$, h=2,3,⋯, we have*

(A22) $$\begin{array}{c} R_{m,n}\left(X_{m},Y_{n}\right)\leq c_{\epsilon ,h}^{\prime \prime }\;{\left(\#X_{m}\;\#Y_{n}\right)}^{1-1/d}. \end{array}$$

*Here, $c_{\epsilon ,h}^{\prime \prime }=O\left(\frac{\epsilon }{h^{d-1}-1}\right)$ depending only on ϵ and h.*

The complexity of $R_{m,n}$’s behavior and the need to pursue the proof encouraged us to explore the smoothness condition for $R_{m,n}$. In fact, this is where both subadditivity and superadditivity for the FR statistic are used together and become more important.

**Lemma** **A10** (Smoothness for $R_{m,n}$)**.** *Given observations of*

$$X_{m}:=\left(X_{m^{\prime }},X_{m^{\prime \prime }}\right)=\left\{X_{1},\cdots ,X_{m^{\prime }},X_{m^{\prime }+1},\cdots ,X_{m}\right\},$$

*where $m^{\prime }+m^{\prime \prime }=m$ and $Y_{n}:=\left(Y_{n^{\prime }},Y_{n^{\prime \prime }}\right)=\left\{Y_{1},\cdots ,Y_{n^{\prime }},Y_{n^{\prime }+1},\cdots ,Y_{n}\right\}$, where $n^{\prime }+n^{\prime \prime }=n$, denote $R_{m,n}\left(X_{m},Y_{n}\right)$ as before, the number of edges of $\mathrm{MST}(X_{m},Y_{n})$ which connect a point of $X_{m}$ to a point of $Y_{n}$. Then, for given integer h≥2, for all $\left(X_{n},Y_{m}\right)\in {\left[0,1\right]}^{d}$, $\epsilon \geq h^{2}\;\delta_{m,n}^{h}$ where $\delta_{m,n}^{h}=O\left(h^{d-1}{\left(m+n\right)}^{1/d}\right)$, we have*

(A23) $$\begin{array}{c} P\left(|R_{m,n}\left(X_{m},Y_{n}\right)-R_{m^{\prime },n^{\prime }}\left(X_{m^{\prime }},Y_{n^{\prime }}\right)|\leq {\tilde{c}}_{\epsilon ,h}\;{\left(\#X_{m^{\prime \prime }}\;\#Y_{n^{\prime \prime }}\right)}^{1-1/d}\right)\\ \;\;\;\geq 1-\frac{2h\;\delta_{m,n}^{h}}{\epsilon }, \end{array}$$

*where ${\tilde{c}}_{\epsilon ,h}=O\left(\frac{\epsilon }{h^{d-1}-1}\right)$.*

*Remark:* Using Lemma A10, we can imply the continuty property, i.e., for all observations $\left(X_{m},Y_{n}\right)$ and $\left(X_{m^{\prime }},Y_{n^{\prime }}\right)$, with at least probability 2g(ϵ)−1, one obtains

(A24) $$\begin{array}{c} \begin{array}{c} \left|R_{m,n}\left(X_{m},Y_{n}\right)-R_{m^{\prime },n^{\prime }}\left(X_{m^{\prime }},Y_{n^{\prime }}\right)\right|\\ \;\;\leq c_{\epsilon ,h}^{*}\;{\left(\#\left(X_{m}\Delta \;X_{m^{\prime }}\right)\;\#\left(Y_{n}\Delta \;Y_{n^{\prime }}\right)\right)}^{1-1/d}, \end{array} \end{array}$$

for given ϵ>0, $c_{\epsilon ,h}^{*}=O\left(\frac{\epsilon }{h^{d-1}-1}\right)$, h≥2. Here, $X_{m}\Delta \;X_{m^{\prime }}$ denotes symmetric difference of observations $X_{m}$ and $X_{m^{\prime }}$.

The path to approach the assertions (11) and (12) proceeds via semi-isoperimentic inequality for the $R_{m,n}$ involving the Hamming distance.

**Lemma** **A11** (Semi-Isoperimetry)**.** *Let μ be a measure on ${\left[0,1\right]}^{d}$; $\mu^{n}$ denotes the product measure on space ${\left({\left[0,1\right]}^{d}\right)}^{n}$. In addition, let $M_{e}$ denotes a median of $R_{m,n}$. Set*

(A25) $$\begin{array}{c} A:=\left\{X_{m}\in {\left({\left[0,1\right]}^{d}\right)}^{m},Y_{n}\in {\left({\left[0,1\right]}^{d}\right)}^{n};R_{m,n}\left(X_{m},Y_{n}\right)\leq M_{e}\right\}. \end{array}$$

*Following the notations in [17], $H\left(x,x^{\prime }\right)=\#\left\{i,x_{i}\neq x_{i}^{\prime }\right)$ and $\phi_{A}\left(x^{\prime }\right)+\phi_{A}\left(y^{\prime }\right)=\min\left\{H\left(x,x^{\prime }\right)+H\left(y,y^{\prime }\right):x,y\in A\right\}$ and $\phi_{A}\left(x^{\prime }\right)\;\phi_{A}\left(y^{\prime }\right)=\min\left\{H\left(x,x^{\prime }\right)\;H\left(y,y^{\prime }\right):x,y\in A\right\}$. Then,*

(A26) $$\begin{array}{c} \mu^{m+n}\left(\left\{x^{\prime }\in {\left({\left[0,1\right]}^{d}\right)}^{m},y^{\prime }\in {\left({\left[0,1\right]}^{d}\right)}^{n}:\phi_{A}\left(x^{\prime }\right)\;\phi_{A}\left(y^{\prime }\right)\geq t\right\}\right)\\ \;\;\leq 4\exp\left(\frac{-t}{8(m+n)}\right). \end{array}$$

Now, we continue by providing the proof of Theorem 5. Recall (A25) and denote

$$\begin{array}{c} \begin{array}{c} F_{x}:=\left\{x_{i},i=1,\cdots ,m,x_{i}=x_{i}^{\prime }\right\},\\ F_{y}:=\left\{y_{j},j=1,\cdots ,n,y_{j}=y_{j}^{\prime }\right\},\\ \mathrm{and}\\ G_{x}:=\left\{x_{i},i=1,\cdots ,m,x_{i}\neq x_{i}^{\prime }\right\},\\ G_{y}:=\left\{y_{j},j=1,\cdots ,n,y_{j}\neq y_{j}^{\prime }\right\}. \end{array} \end{array}$$

In addition, for given integer *h*, define events B, $B^{\prime }$ by

$$\begin{array}{c} \begin{array}{c} B:=\left\{|R_{m,n}\left(X_{m}^{\prime },Y_{n}^{\prime }\right)-R\left(F_{x},F_{y}\right)|\leq c_{\epsilon ,h}{\left(\#G_{x}\;\#G_{y}\right)}^{1-1/d}\right\},\\ B^{\prime }:=\left\{|R\left(F_{x},F_{y}\right)-R_{m,n}\left(X_{m},Y_{n}\right)|\leq c_{\epsilon ,h}{\left(\#G_{x}\;\#G_{y}\right)}^{1-1/d}\right\}, \end{array} \end{array}$$

where $c_{\epsilon ,h}$ is a constant. By virtue of smoothness property, Lemma A10, for $\epsilon \geq h^{2}\delta_{m,n}^{h}$, we know P(B)≥2g(ϵ)−1 and $P\left(B^{\prime }\right)\geq 2g\left(\epsilon \right)-1$. On the other hand, we have

$$\begin{array}{c} R_{m,n}\left(X_{m}^{\prime },Y_{n}^{\prime }\right)\leq |R_{m,n}\left(X_{m}^{\prime },Y_{n}^{\prime }\right)-R\left(F_{x},F_{y}\right)|\\ \;\;+|R\left(F_{x},F_{y}\right)-R_{m,n}\left(X_{m},Y_{n}\right)|+R_{m,n}\left(X_{m},Y_{n}\right).\\ \;=|\varpi^{\prime }|+|\varpi |+R_{m,n}\left(X_{m},Y_{n}\right)\;\;\;\left(\mathrm{say}\right). \end{array}$$

Moreover, $P(R_{m,n}\left(X_{m},Y_{n}\right)\leq M_{e})\geq 1/2$. Therefore, we can write

(A27) $$\begin{array}{c} 1/2\leq P\left(R_{m,n}\left(X_{m}^{\prime },Y_{n}^{\prime }\right)\leq M_{e}+|\varpi^{\prime }\left|+|\varpi \right|\right)\\ \leq P\left(R_{m,n}\left(X_{m}^{\prime },Y_{n}^{\prime }\right)\leq M_{e}+|\varpi^{\prime }|+|\varpi ||\;B\cap B^{\prime }\right)P\left(B\cap B^{\prime }\right)\\ +P(B^{c}\cup B^{\prime c}). \end{array}$$

Thus, we obtain

$$\begin{array}{c} \begin{array}{c} P\left(R_{m,n}\left(X_{m}^{\prime },Y_{n}^{\prime }\right)\leq M_{e}+4\epsilon \;{\left(\#G_{x}\;\#G_{y}\right)}^{1-1/d}\right)\\ \;\;\geq \left(1/2-1+P(B\cap B^{\prime })\right)/P\left(B\cap B^{\prime }\right)\\ \;\;\;=1-\left({\left(2\;P(B\cap B^{\prime })\right)}^{-1}\right). \end{array} \end{array}$$

Note that $P\left(B\cap B^{\prime }\right)=P\left(B\right)\;P\left(B^{\prime }\right)\geq {\left(2\;g(\epsilon )-1\right)}^{2}$. Now, we easily claim that

(A28) $$1-\left({\left(2\;P(B\cap B^{\prime })\right)}^{-1}\right)\geq 1-\left({\left(2\;{\left(2\;g\left(\epsilon \right)-1\right)}^{2}\right)}^{-1}\right).$$

Thus,

$$\begin{array}{c} P\left(R_{m,n}\left(X_{m}^{\prime },Y_{n}^{\prime }\right)\leq M_{e}+4\epsilon \;{\left(\#G_{x}\;\#G_{y}\right)}^{1-1/d}\right)\geq 1-\left({\left(2\;{\left(2\;g\left(\epsilon \right)-1\right)}^{2}\right)}^{-1}\right). \end{array}$$

On the other word, calling $\phi_{A}\left(x^{\prime }\right)$ and $\phi_{A}\left(y^{\prime }\right)$ in Lemma A11, we get

(A29) $$\begin{array}{c} \begin{array}{c} P\left(R_{m,n}\left(X_{m}^{\prime },Y_{n}^{\prime }\right)\leq M_{e}+4\epsilon \;{\left(\phi_{A}\left(x^{\prime }\right)\;\phi_{A}\left(y^{\prime }\right)\right)}^{1-1/d}\right)\geq 1-\left({\left(2\;{\left(2\;g\left(\epsilon \right)-1\right)}^{2}\right)}^{-1}\right). \end{array} \end{array}$$

Furthermore, denote event

$$C:=\left\{R_{m,n}\left(X_{m}^{\prime },Y_{n}^{\prime }\right)\leq M_{e}+4\epsilon \;{\left(\phi_{A}\left(x^{\prime }\right)\;\phi_{A}\left(y^{\prime }\right)\right)}^{1-1/d}\right\}.$$

Then, we have

(A30) $$\begin{array}{c} P\left(R_{m,n}\left(X_{m},Y_{n}\right)\geq M_{e}+t\right)=\mu^{m+n}\left(R_{m,n}\left(X_{m}^{\prime },Y_{n}^{\prime }\right)\geq M_{e}+t\right)\\ \;=\mu^{m+n}\left(\left(R_{m,n}\left(X_{m}^{\prime },Y_{n}^{\prime }\right)\geq M_{e}+t\right)|C\right)P\left(C\right)\\ \;\;+\mu^{m+n}\left(\left(R_{m,n}\left(X_{m}^{\prime },Y_{n}^{\prime }\right)\geq M_{e}+t\right)|C^{c}\right)P\left(C^{c}\right)\\ \;\leq \mu^{m+n}\left({\left(\phi_{A}\left(x^{\prime }\right)\;\phi_{A}\left(y^{\prime }\right)\right)}^{1-1/d}\geq \frac{t}{4\epsilon }\right)P\left(C\right)\\ \;\;+\mu^{m+n}\left(\left(R_{m,n}\left(X_{m}^{\prime },Y_{n}^{\prime }\right)\geq M_{e}+t\right)|C^{c}\right)P\left(C^{c}\right).\\ \mathrm{Using}\;\;P\left(C\right)=1-P\left(C^{c}\right)\\ \;=\mu^{m+n}\left({\left(\phi_{A}\left(x^{\prime }\right)\;\phi_{A}\left(y^{\prime }\right)\right)}^{1-1/d}\geq \frac{t}{4\epsilon }\right)\\ \;\;+P\left(C^{c}\right)\{\mu^{m+n}\left(\left(R_{m,n}\left(X_{m}^{\prime },Y_{n}^{\prime }\right)\geq M_{e}+t\right)|C^{c}\right)\\ \;\;\;-\mu^{m+n}\left({\left(\phi_{A}\left(x^{\prime }\right)\;\phi_{A}\left(y^{\prime }\right)\right)}^{1-1/d}\geq \frac{t}{4\epsilon }\right)\}. \end{array}$$

Define set $K_{t}=\left\{{\left(\phi_{A}\left(x^{\prime }\right)\;\phi_{A}\left(y^{\prime }\right)\right)}^{1-1/d}\geq \frac{t}{4\epsilon }\right\}$, so

$$\begin{array}{c} \begin{array}{c} \mu^{m+n}\left(R_{m,n}\left(X_{m}^{\prime },Y_{n}^{\prime }\right)\geq M_{e}+t|C^{c}\right)\\ =\mu^{m+n}\left(R_{m,n}\left(X_{m}^{\prime },Y_{n}^{\prime }\right)\geq M_{e}+t|C^{c},K_{t}\right)\mu^{m+n}\left(K_{t}\right)+\mu^{m+n}\left(\left(R_{m,n}\left(X_{m}^{\prime },Y_{n}^{\prime }\right)\geq M_{e}+t\right)|C^{c},K_{t}^{c}\right)\mu^{m+n}\left(K_{t}^{c}\right). \end{array} \end{array}$$

Since

$$\mu^{m+n}\left(R_{m,n}\left(X_{m}^{\prime },Y_{n}^{\prime }\right)\geq M_{e}+t|C^{c},K_{t}\right)=1,$$

and

$$\begin{array}{c} \begin{array}{c} \mu^{m+n}\left(R_{m,n}\left(X_{m}^{\prime },Y_{n}^{\prime }\right)\geq M_{e}+t|C^{c},K_{t}^{c}\right)=\mu^{m+n}\left(R_{m,n}\left(X_{m}^{\prime },Y_{n}^{\prime }\right)\geq M_{e}+t\right). \end{array} \end{array}$$

Consequently, from (A30), one can write

(A31) $$\begin{array}{c} \begin{array}{c} P\left(R_{m,n}\left(X_{m},Y_{n}\right)\geq M_{e}+t\right)\\ \;\;\leq \mu^{m+n}\left({\left(\phi_{A}\left(x^{\prime }\right)\;\phi_{A}\left(y^{\prime }\right)\right)}^{1-1/d}\geq \frac{t}{4\epsilon }\right)\\ \;+P\left(C^{c}\right)\left\{\mu^{m+n}\left(R_{m,n}\left(X_{m}^{\prime },Y_{n}^{\prime }\right)\geq M_{e}+t\right)\mu^{m+n}\left(K_{t}^{c}\right)\right\}\\ \;\;\leq \mu^{m+n}\left({\left(\phi_{A}\left(x^{\prime }\right)\;\phi_{A}\left(y^{\prime }\right)\right)}^{1-1/d}\geq \frac{t}{4\epsilon }\right)\\ \;+\left({\left(2\;{\left(2\;g\left(\epsilon \right)-1\right)}^{2}\right)}^{-1}\right)P\left(R_{m,n}\left(X_{m},Y_{n}\right)\geq M_{e}+t\right). \end{array} \end{array}$$

The last inequality implies by owing to (A29) and $\mu^{m+n}\left(K_{t}^{c}\right)\leq 1$. For $g\left(\epsilon \right)\geq 1/2+1/\left(2\sqrt{2}\right)$, we have

$$1-\left({\left(2\;{\left(2\;g\left(\epsilon \right)-1\right)}^{2}\right)}^{-1}\right)\geq 0,$$

or equivalently this holds true when $\epsilon \geq \left(2h\sqrt{2}\;\delta_{m,n}^{h}\right)/\left(\sqrt{2}-1\right)$. Furthermore, for h≥7, we have

(A32) $$h^{2}\delta_{m,n}^{h}\geq \left(2h\sqrt{2}\;\delta_{m,n}^{h}\right)/\left(\sqrt{2}-1\right),$$

therefore $P\left(R_{m,n}\left(X_{m},Y_{n}\right)\geq M_{e}+t\right)$ is less than and equal to

(A33) $${\left(1-\left({\left(2\;{\left(2\;g\left(\epsilon \right)-1\right)}^{2}\right)}^{-1}\right)\right)}^{-1}\mu^{m+n}\left({\left(\phi_{A}\left(x^{\prime }\right)\;\phi_{A}\left(y^{\prime }\right)\right)}^{1-1/d}\geq \frac{t}{4\epsilon }\right).$$

By virtue of Lemma A11, finally we obtain

(A34) $$\begin{array}{c} P\left(R_{m,n}\left(X_{m},Y_{n}\right)\geq M_{e}+t\right)\leq 4\;{\left(1-\left({\left(2\;{\left(2\;g\left(\epsilon \right)-1\right)}^{2}\right)}^{-1}\right)\right)}^{-1}\exp\left(\frac{-t^{d/(d-1)}}{8{\left(4\epsilon \right)}^{d/d-1}\left(m+n\right)}\right). \end{array}$$

Similarly, we can derive the same bound on $P\left(R_{m,n}\left(X_{m},Y_{n}\right)\leq M_{e}-t\right)$, so we obtain

(A35) $$\begin{array}{c} P\left(|R_{m,n}-M_{e}|\geq t\right)\leq C_{m,n}^{\prime }\left(\epsilon ,h\right)\exp\left(\frac{-t^{d/(d-1)}}{8{\left(4\epsilon \right)}^{d/(d-1)}\left(m+n\right)}\right), \end{array}$$

where

(A36) $$\begin{array}{c} C_{m,n}^{\prime }\left(\epsilon ,h\right)=8{\left(1-2^{-1}\;{\left(1-\frac{2h\;\;O\left(h^{d-1}{\left(m+n\right)}^{1/d}\right)}{\epsilon }\right)}^{-2}\right)}^{-1}. \end{array}$$

We will analyze (A35) together with Theorem 6. The next lemma will be employed in Theorem 6’s proof.

**Lemma** **A12** (Deviation of the Mean and Median)**.** *Consider $M_{e}$ as a median of $R_{m,n}$. Then, for $\epsilon \geq h^{2}\delta_{m,n}^{h}$ and given h≥7, we have*

(A37) $$|E\left[R_{m,n}\left(X_{m},Y_{n}\right)\right]-M_{e}|\leq C_{m,n}\left(\epsilon ,h\right)\;{\left(m+n\right)}^{(d-1)/d},$$

*where $C_{m,n}\left(\epsilon ,h\right)$ is a constant depending on ϵ, h, m, and n by*

(A38) $$\begin{array}{c} C_{m,n}\left(\epsilon ,h\right)=C\;{\left(1-\left({\left(2\;{\left(2\;g\left(\epsilon \right)-1\right)}^{2}\right)}^{-1}\right)\right)}^{-1}, \end{array}$$

where *C* is a constant and

$$\delta_{m,n}^{h}=O\left(h^{d-1}{\left(m+n\right)}^{1/d}\right),\;\;\mathrm{and}\;\;g\left(\epsilon \right)=1-\frac{h\;\delta_{m,n}^{h}}{\epsilon }.$$

We conclude this part by pursuing our primary intension which has been the Theorem 6’s proof. Observe from Theorem 5, (11) that

$$\begin{array}{c} \begin{array}{c} P(|R_{m,n}-E\left[R_{m,n}\right]|\geq t+C_{m,n}\left(\epsilon ,l\right)\left({m+n)}^{(d-1)/d}\right)\\ \;\leq P(|R_{m,n}-M_{e}|+|E\left[R_{m,n}\right]-M_{e}|\\ \;\;\;\geq t+C_{m,n}\left(\epsilon ,l\right)\left({m+n)}^{(d-1)/d}\right)\\ \;\leq P\left(|R_{m,n}-M_{e}|\geq t\right)\\ \leq 8\;{\left(1-\left({\left(2\;{\left(2\;g\left(\epsilon \right)-1\right)}^{2}\right)}^{-1}\right)\right)}^{-1}\;\exp\left(\frac{-t^{d/(d-1)}}{8{\left(4\epsilon \right)}^{d/d-1}\left(m+n\right)}\right). \end{array} \end{array}$$

Note that the last bound is derived by (11). The rest of the proof is as the following: When $t\geq 2C_{m,n}{\left(\epsilon ,h\right)(m+n)}^{(d-1)/d}$, we use

$$(t-C_{m,n}\left(\epsilon ,h\right){\left({m+n)}^{(d-1)/d}\right)}^{d/(d-1)}\geq {\left(t/2\right)}^{d/(d-1)}.$$

Therefore, it turns out that

(A39) $$\begin{array}{c} P\left(|R_{m,n}-E\left[R_{m,n}\right]|\geq t\right)\\ \leq 8\;{\left(1-\left({\left(2\;{\left(2\;g\left(\epsilon \right)-1\right)}^{2}\right)}^{-1}\right)\right)}^{-1}\exp\left(\frac{-t^{d/(d-1)}}{8{\left(8\epsilon \right)}^{d/(d-1)}\left(m+n\right)}\right). \end{array}$$

In other words, there exist constants $C_{m,n}^{\prime }\left(\epsilon ,h\right)$ depending on m,n, ϵ, and *h* such that

(A40) $$\begin{array}{c} P\left(|R_{m,n}-E\left[R_{m,n}\right]|\geq t\right)\leq C_{m,n}^{\prime }\left(\epsilon ,h\right)\exp\left(\frac{-{\left(t/\left(2\epsilon \right)\right)}^{d/(d-1)}}{\left(m+n\right)\;\tilde{C}}\right), \end{array}$$

where $\tilde{C}=8{\left(4\right)}^{d/(d-1)}$.

To verify the behavior of bound (A40) in terms of ϵ, observe (A35) first; it is not hard to see that this function is decreasing in ϵ. However, the function

$$\exp\left(\frac{-{\left(t/\left(2\epsilon \right)\right)}^{d/(d-1)}}{\left(m+n\right)\tilde{C}}\right)$$

increases in ϵ. Therefore, one can not immediately infer that the bound in (12) is monotonic with respect to ϵ. For fixed N=n+m, *d*, and *h*, the first and second derivatives of the bound (12) with respect to ϵ are quite complicated functions. Thus, deriving an explicit optimal solution for the minimization problem with the objective function (12) is not feasible. However, in the sequel, we discuss that under conditions when *t* is not much larger than N=m+n, this bound becomes convex with respect to ϵ. Set

(A41) $$\begin{array}{c} K\left(\epsilon \right)=C_{m,n}^{\prime }\left(\epsilon ,h\right)\;\exp\left(\frac{-B(t)}{\epsilon^{d/(d-1)}}\right), \end{array}$$

where $C_{m,n}^{\prime }$ is given in (10) and

$$B\left(t\right)=\frac{t^{d/(d-1)}}{8\;{\left(8\right)}^{d/(d-1)}\left(N\right)}.$$

By taking the derivative with respect to ϵ, we have

(A42) $$\begin{array}{c} \frac{dK(\epsilon )}{d\epsilon }=K\left(\epsilon \right)\left(\frac{d}{d\epsilon }\left(\log C_{m,n}^{\prime }\right)+\frac{B(t)\;d/(d-1)}{\epsilon^{\left(2d-1)/(d-1\right)}}\right), \end{array}$$

where

(A43) $$\begin{array}{c} \frac{d}{d\epsilon }\left(\log C_{m,n}^{\prime }\right)=\frac{-4\;a_{h}\;\epsilon }{\left(\epsilon -2a_{h}\right)\left(8a_{h}^{2}-8\epsilon a_{h}+\epsilon^{2}\right)}, \end{array}$$

where $a_{h}=h\delta_{m,n}^{h}$. The second derivative K(ϵ) with respect to ϵ after simplification is given as

(A44) $$\begin{array}{c} \frac{d^{2}}{d\epsilon^{2}}K\left(\epsilon \right)={\left(\frac{-4\;a_{h}\;\epsilon }{\left(\epsilon -2a_{h}\right)\left(8a_{h}^{2}-8\epsilon a_{h}+\epsilon^{2}\right)}+\frac{B\left(t\right)\;\bar{d}}{\epsilon^{\bar{d}+1}}\right)}^{2}\\ +K\left(\epsilon \right)\left(\frac{8a_{h}\;\left(8a_{h}^{3}+\epsilon^{2}\left(\epsilon -5a_{h}\right)\right)}{{\left(8a_{h}^{2}-8a_{h}\epsilon +\epsilon^{2}\right)}^{2}{\left(\epsilon -2a_{h}\right)}^{2}}-\frac{B\left(t\right)\bar{d}\left(\bar{d}+1\right)}{\epsilon^{\bar{d}+2}}\right), \end{array}$$

where $\bar{d}=d/\left(d-1\right)$. The first term in (A44) and K(ϵ) are non-negative, so K(ϵ) is convex if the second term in the second line of (A44) is non-negative. We know that $\epsilon \geq h^{2}\delta_{m,n}^{h}=h\;a_{h}$, when h=7, we can parameterize ϵ by setting it equal to $\gamma a_{h}$, where γ≥7. After simplification, K(ϵ) is convex if

(A45) $$\begin{array}{c} a_{h}^{\bar{d}-1}\left(\gamma^{\bar{d}-1}+3\gamma^{\bar{d}-2}\right)+B\left(t\right)\bar{d}\left(\bar{d}+1\right)\\ \times \{a_{h}^{-1}\left(-32\gamma^{-6}+64\gamma^{-5}-48\gamma^{-4}+8\gamma^{-3}-\frac{7}{2}\gamma^{-2}+2\gamma^{-1}-\frac{1}{8}\right)\\ +a_{h}^{-2}\left(32\gamma^{-6}-64\gamma^{-5}+40\gamma^{-4}+8\gamma^{-3}+\frac{1}{2}\gamma^{-2}\right)\}\geq 0. \end{array}$$

This is implied if

(A46) $$\begin{array}{c} 0\leq B\left(t\right)\bar{d}\left(\bar{d}+1\right)\;a_{h}^{-1}\\ \times \left(-32\gamma^{-6}+64\gamma^{-5}-48\gamma^{-4}+8\gamma^{-3}-\frac{7}{2}\gamma^{-2}+2\gamma^{-1}-\frac{1}{8}\right), \end{array}$$

such that γ≥7. One can easily check that, as γ→∞, then (A46) tends to $-\frac{1}{8}B\left(t\right)\bar{d}\left(\bar{d}+1\right)\;a_{h}^{-1}$. This term can be negligible unless we have *t* that is much larger than N=m+n with the threshold depending on *d*. Here, by setting $B\left(t\right)/a_{h}=1$, a rough threshold $t=O\left(7^{d-1}{\left(m+n\right)}^{1-1/d^{2}}\right)$ depending on *d*, m+n is proposed. Therefore, minimizing (A35) and (A40) with respect to ϵ when optimal h=7 is a convex optimization problem. Denote $\epsilon^{*}$ the solution of the convex optimization problem (9). By plugging optimal *h* (h=7) and ϵ ($\epsilon =\epsilon^{*})$ in (A35) and (A40), we derive (11) and (12), respectively.

In this appendix, we also analyze the bound numerically. By simulation, we observed that lower *h* i.e., h=7 is the optimal value experimentally. Indeed, this can be verified by Theorem 11’s proof. We address the reader to Lemma A8 in Appendix D and Appendix E where, as *h* increases, the lower bound for the probability increases too. In other words, for fixed N=m+n and *d*, the lowest *h* implies the maximum bound in (A92). For this, we set h=7 in our experiments. We vary the dimension *d* and sample size N=m+n in relatively large and small ranges. In Table A1, we solve (9) for various values of *d* and N=m+n. We also compute the lower bound for ϵ i.e., $7^{d+1}N^{1/d}$ per experiment. In Table A1, we observe that as we have higher dimension the optimal value $\epsilon^{*}$ equals the ϵ lower bound $h^{d+1}N^{1/d}$, but this is not true for smaller dimensions with even relatively large sample size.

**Table A1:** *d*, *N*, $\epsilon^{*}$ are dimension, total sample size m+n, and optimal ϵ for the bound in (12). The column $h^{d+1}N^{1/d}$ represents approximately the lower bound for ϵ which is our constraint in the minimization problem and our assumption in Theorems 5 and 6. Here, we set h=7.

Concentration Bound (11)					
d	N=m+n	$\epsilon^{*}$	$t_{0}$	$h^{d+1}N^{1/d}$	**Optimal (11)**
2	$10^{3}$	$1.1424\times 10^{4}$	$2\times 10^{7}$	$1.0847\times 10^{4}$	0.3439
4	$10^{4}$	$1.7746\times 10^{5}$	$3\times 10^{10}$	168,070	0.0895
5	550	$4.7236\times 10^{5}$	$10^{10}$	$4.1559\times 10^{5}$	0.9929
6	$10^{4}$	$3.8727\times 10^{6}$	$2\times 10^{12}$	$3.8225\times 10^{6}$	0.1637
8	1200	$9.7899\times 10^{7}$	$12\times 10^{12}$	$9.7899\times 10^{7}$	0.7176
10	3500	$4.4718\times 10^{9}$	$2\times 10^{15}$	$4.4718\times 10^{9}$	0.4795
15	$10^{8}$	$1.1348\times 10^{14}$	$10^{24}$	$1.1348\times 10^{14}$	0.9042

To validate our proposed bound in (12), we again set h=7 and for d=4,5,7 we ran experiments with sample sizes N=m+n=9000,1100,140, respectively. Then, we solved the minimization problem to derive optimal bound for *t* in the range $10^{10}\left[1,3\right]$. Note that we chose this range to have a non-trivial bound for all three curves; otherwise, the bounds partly become one. Figure A1 shows that when *t* increases in the given range, the optimal curves approach zero.

To prove the Theorem 7 in the concentration of $R_{m,n}$, Theorem 6, let

$$\delta =C_{m,n}^{\prime }\left(\epsilon^{*}\right)\exp\left(\frac{-{\left(t/\left(2\epsilon^{*}\right)\right)}^{d/(d-1)}}{\left(m+n\right)\;\tilde{C}}\right),$$

this implies $t=O(\epsilon^{*}\;{\left(m+n\right)}^{(d-1)/d}\left(\log{\left(C_{m,n}^{\prime }\left(\epsilon^{*}\right)/\delta )\right)}^{(d-1)/d}\right)$ and the proofs are completed.

## Appendix E. Additional Proofs

Lemma A3: Let g(x) be a density function with support ${\left[0,1\right]}^{d}$ and belong to the Hölder class $\Sigma_{d}\left(\eta ,L\right)$, 0<η≤1, expressed in Definition 1. In addition, assume that P(x) is a η-Hölder smooth function, such that its absolute value is bounded from above by some constants *c*. Define the quantized density function with parameter *l* and constants $\phi_{i}$ as

(A47) $$\begin{array}{c} \hat{g}\left(x\right)=\sum_{i=1}^{M}\phi_{i}1\left\{x\in Q_{i}\right\},\;\;\;\mathrm{where}\;\phi_{i}=l^{d}\;\int_{Q_{i}}g\left(x\right)\;dx, \end{array}$$

and $M=l^{d}$ and $Q_{i}=\{x,x_{i}:∥x-x_{i}∥<l^{-d}\}$. Then,

(A48) $$\begin{array}{c} \int ∥\left(g\left(x\right)-\hat{g}\left(x\right)\right)P\left(x\right)∥\;dx\leq O\left(l^{-d\eta }\right). \end{array}$$

**Proof.** By the mean value theorem, there exist points $\epsilon_{i}\in Q_{i}$ such that

$$\begin{array}{c} \phi_{i}=l^{d}\;\int_{Q_{i}}g\left(x\right)\;dx=g\left(\epsilon_{i}\right). \end{array}$$

Using the fact that $g\in \Sigma_{d}\left(\eta ,L\right)$ and P(x) is a bounded function, we have

$$\begin{array}{c} \begin{array}{cc} \int ∥g\left(x\right)-\hat{g}\left(x\right))P\left(x\right)∥\;dx & =\sum_{i=1}^{M}\int_{Q_{i}}∥\left(g\left(x\right)-\Phi_{i}\right)P\left(x\right)∥dx\\ & =\sum_{i=1}^{M}\int_{Q_{i}}∥\left(g\left(x\right)-g\left(\epsilon_{i}\right)\right)P\left(x\right)∥dx\\ & \leq c\;L\sum_{i=1}^{M}\int_{Q_{i}}∥x-\epsilon_{i}{∥}^{\eta }\;dx. \end{array} \end{array}$$

Here, *L* is the Hölder constant. As $x,\epsilon_{i}\in Q_{i}$, a sub-cube with edge length $l^{-1}$, then $∥x-\epsilon_{i}{∥}^{\eta }=O\left(l^{-d\eta }\right)$ and $\sum_{i=1}^{M}\int_{Q_{i}}\;dx=1$. This concludes the proof. □

Lemma A4: Let Δ(x,S) denote the degree of vertex x∈S in the MST over set $S\subset R^{d}$ with the *n* number of vertices. For given function P(x,x), one yields

(A49) $$\int P\left(x,x\right)g\left(x\right)E\left[\Delta \left(x,S\right)\right]\;dx=2\;\int P\left(x,x\right)g\left(x\right)\;dx+\varsigma_{\eta }\left(l,n\right),$$

where for constant η>0,

(A50) $$\begin{array}{c} \varsigma_{\eta }\left(l,n\right)=\left(O\left(l/n\right)-2\;l^{d}/n\right)\int g\left(x\right)P\left(x,x\right)\;dx+O\left(l^{-d\eta }\right). \end{array}$$

**Proof.** Recall notations in Lemma A3 and

$$\begin{array}{c} |\int g\left(x\right)P\left(x\right)\;dx-\int \hat{g}\left(x\right)P\left(x\right)\;dx|\leq \int |\left(g\left(x\right)-\hat{g}\left(x\right)\right)P\left(x\right)|\;dx. \end{array}$$

Therefore, by substituting $\hat{g}$, defined in (A47), into *g* with considering its error, we have

(A51) $$\begin{array}{c} \int P(x,x)g(x)E[\Delta (x,S)]\;dx\\ \;\;=\int P\left(x,x\right)E\left[\Delta \left(x,S\right)\right]\sum_{i=1}^{M}\phi_{i}1\left\{x\in Q_{i}\right\}\;dx+O\left(l^{-d\eta }\right)\\ \;\;=\sum_{i=1}^{M}\phi_{i}\int_{Q_{i}}P\left(x,x\right)E\left[\Delta \left(x,S\right)\right]\;dx+O\left(l^{-d\eta }\right). \end{array}$$

Here, $Q_{i}$ represents as before in Lemma A3, so the RHS of (A51) becomes

(A52) $$\begin{array}{c} \begin{array}{c} \sum_{i=1}^{M}\phi_{i}\int_{Q_{i}}P\left(x,x\right)E\left[\Delta \left(x,S\cap Q_{i}\right)\right]\;dx+\sum_{i=1}^{M}\phi_{i}\int_{Q_{i}}P\left(x,x\right)O\left(l^{1-d}/n\right)+O\left(l^{-d\eta }\right)\\ \;=\sum_{i=1}^{M}\phi_{i}P\left(x_{i},x_{i}\right)\frac{1}{M}\int_{Q_{i}}M\;E\left[\Delta \left(x,S\cap Q_{i}\right)\right]\;dx+\sum_{i=1}^{M}\phi_{i}\int_{Q_{i}}P\left(x,x\right)O\left(l^{1-d}/n\right)+2\;O\left(l^{-d\eta }\right). \end{array} \end{array}$$

Now, note that $\int_{Q_{i}}M\;E\left[\Delta \left(x,S\cap Q_{i}\right)\right]\;dx$ is the expectation of $E[\Delta \left(x,S\cap Q_{i}\right)]$ over the nodes in $Q_{i}$, which is equal to $2-\frac{2}{k_{i}}$, where $k_{i}=\frac{n}{M}$. Consequently, we have

(A53) $$\begin{array}{c} \int P\left(x,x\right)g\left(x\right)E\left[\Delta \left(x,S\right)\right]\;dx=\left(2-\frac{2\;M}{n}\right)\sum_{i=1}^{M}\phi_{i}\;P\left(x_{i},x_{i}\right)\frac{1}{M}+O\left(\frac{l^{1-d}}{n}\right)\sum_{i=1}^{M}\phi_{i}\;P\left(x_{i},x_{i}\right)+3\;O\left(l^{-d\eta }\right)\\ \;\;=2\int g\left(x\right)P\left(x,x\right)\;dx+5\;O\left(l^{-d\eta )}\right)+M\;\left(O\left(\frac{l^{1-d}}{n}\right)-\left(\frac{2}{n}\right)\right)\int g\left(x\right)P\left(x,x\right)\;dx. \end{array}$$

This gives the assertion (A49). □

Lemma A5: Assume that, for given *k*, $g_{k}\left(x\right)$ is a bounded function belong to $\Sigma_{d}\left(\eta ,L\right)$. Let $P:R^{d}\times R^{d}\mapsto \left[0,1\right]$ be a symmetric, smooth, jointly measurable function, such that, given *k*, for almost every $x\in R^{d}$, P(x,.) is measurable with x a Lebesgue point of the function $g_{k}\left(.\right)P\left(x,.\right)$. Assume that the first derivative *P* is bounded. For each *k*, let $Z_{1}^{k},Z_{2}^{k},\cdots ,Z_{k}^{k}$ be independent *d*-dimensional variable with common density function $g_{k}$. Set $Z_{k}=\left\{Z_{1}^{k},Z_{2}^{k}\cdots ,Z_{k}^{k}\right\}$ and $Z_{k}^{x}=\left\{x,Z_{2}^{k},Z_{3}^{k}\cdots ,Z_{k}^{k}\right\}$. Then,

(A54) $$\begin{array}{c} E\left[\sum_{j=2}^{k}P\left(x,Z_{j}^{k}\right)1\left\{\left(x,Z_{j}^{k}\right)\in MST\left(Z_{k}^{x}\right)\right\}\right]\\ \;=P\left(x,x\right)\;E\left[\Delta (x,Z_{k}^{x})\right]+\left\{O\left(k^{-\eta /d}\right)+O\left(k^{-1/d}\right)\right\}. \end{array}$$

**Proof.** Let $B\left(x,r\right)=\{y:∥y-x{∥}_{d}\leq r\}$. For any positive K, we can obtain:

(A55) $$\begin{array}{c} E\sum_{j=2}^{k}|P\left(x,Z_{j}^{k}\right)-P\left(x,x\right)|1\left\{Z_{j}^{k}\in B\left(x,Kk^{-1/d}\right)\right\}\\ \;=\left(k-1\right)\int_{B\left(x;Kk^{-1/d}\right)}|\left(P\left(x,y\right)g_{k}\left(y\right)-P\left(x,x\right)g_{k}\left(x\right)\right)+P\left(x,x\right)\left(g_{k}\left(x\right)-g_{k}\left(y\right)\right)|\;dy\\ \leq \left(k-1\right)[\int_{B\left(x;Kk^{-1/d}\right)}|\left(P\left(x,y\right)g_{k}\left(y\right)-P\left(x,x\right)g_{k}\left(x\right)\right)|dy+O\left(k^{-\eta /d}\right)V\left(B\left(x,Kk^{-1/d}\right)\right], \end{array}$$

where V is the volume of space B which equals $O(k^{-1})$. Note that the above inequality appears because $g_{k}\left(x\right)\in \Sigma_{d}\left(\eta ,L\right)$ and P(x,x)∈[0,1]. The first order Taylor series expansion of P(x,y) around x is

$$\begin{array}{c} \begin{array}{cc} P(x,y) & =P\left(x,x\right)+P^{\left(1\right)}\left(x,x\right)∥y-x∥+o\left({∥y-x∥}^{2}\right)\\ & =P\left(x,x\right)+O\left(k^{-1/d}\right)+o\left(k^{-2/d}\right). \end{array} \end{array}$$

Then, by recalling the Hölder class, we have

$$\begin{array}{c} \begin{array}{cc} \left|P\left(x,y\right)g_{k}\left(y\right)-P\left(x,x\right)g_{k}\left(x\right)\right| & =|\left(P\left(x,x\right)+O\left(k^{-1/d}\right)\right)\left(g_{k}\left(x\right)+O\left(k^{-\eta /d}\right)\right)-P\left(x,x\right)g_{k}\left(x\right)|\\ & =O\left(k^{-\eta /d}\right)+O\left(k^{-1/d}\right). \end{array} \end{array}$$

Hence, the RHS of (A55) becomes

$$\begin{array}{c} \begin{array}{c} \left(k-1\right)\left[\left(O\left(k^{-\eta /d}\right)+O\left(k^{-1/d}\right)\right)V\left(B\left(x,Kk^{-1/d}\right)\right)+O\left(k^{-\eta /d}\right)V\left(B\left(x,Kk^{-1/d}\right)\right)\right]\\ \;\;\;=\left(k-1\right)\left[O\left(k^{-1-\eta /d}\right)+O\left(k^{-1-1/d}\right)\right]. \end{array} \end{array}$$

The expression in (A54) can be obtained by choice of *K*. □

Lemma A6: Consider the notations and assumptions in Lemma A5. Then,

(A56) $$\begin{array}{c} \left|k^{-1}\underset{\;1\leq i<j\leq k}{\sum \sum }P\left(Z_{i}^{k},Z_{j}^{k}\right)1\{\left(Z_{i}^{k},Z_{j}^{k}\right)\in MST\left(Z_{k}\right)-\int_{R^{d}}P\left(x,x\right)g_{k}\left(x\right)\;dx\right|\\ \;\;\;\leq \varsigma_{\eta }\left(l,k\right)+O\left(k^{-\eta /d}\right)+O\left(k^{-1/d}\right). \end{array}$$

Here, MST(S) denotes the MST graph over nice and finite set $S\subset R^{d}$ and η is the smoothness Hölder parameter. Note that $\varsigma_{\eta }\left(l,k\right)$ is given as before in (A50).

**Proof.** Following notations in [49], let Δ(x,S) denote the degree of vertex x in the MST(S) graph. Moreover, let x be a Lebesgue point of $g_{k}$ with $g_{k}\left(x\right)>0$. In addition, let $Z_{k}^{x}$ be the point process $\left\{x,Z_{2}^{k},Z_{3}^{k},\cdots ,Z_{k}^{k}\right\}$. Now, by virtue of (A55) in Lemma A5, we can write

(A57) $$\begin{array}{c} \begin{array}{c} E\left[\sum_{j=2}^{k}P\left(x,Z_{j}^{k}\right)1\left\{\left(x,Z_{j}^{k}\right)\in MST\left(Z_{k}^{x}\right)\right\}\right]=P\left(x,x\right)\;E\left[\Delta (x,Z_{k}^{x})\right]+\left\{O\left(k^{-\eta /d}\right)+O\left(k^{-1/d}\right)\right\}. \end{array} \end{array}$$

On the other hand, it can be seen that

(A58) $$\begin{array}{c} k^{-1}E\left[\underset{1\leq i<j\leq k}{\sum \sum }P\left(Z_{i}^{k},Z_{j}^{k}\right)1\left\{\left(Z_{i}^{k},Z_{j}^{k}\right)\in MST\left(Z_{k}\right)\right\}\right]\\ \;\;=\frac{1}{2}E\left[\sum_{j=2}^{k}P\left(Z_{1}^{k},Z_{j}^{k}\right)1\left\{\left(Z_{i}^{k},Z_{j}^{k}\right)\in MST\left(Z_{k}\right)\right\}\right]\\ \;\;=\frac{1}{2}\int g_{k}\left(x\right)\;dx\;E\left[\sum_{j=2}^{k}P\left(x,Z_{j}^{k}\right)1\left\{\left(x,Z_{j}^{k}\right)\in MST\left(Z_{k}\right)\right\}\right]. \end{array}$$

Recalling (A57),

(A59) $$\begin{array}{c} =\frac{1}{2}\int g_{k}\left(x\right)P\left(x,x\right)E\left[\Delta (x,Z_{k}^{x})\right]\;dx+O\left(k^{-\eta /d}\right)+O\left(k^{-1/d}\right). \end{array}$$

By virtue of Lemma A4, (A49) can be substituted into expression (A59) to obtain (A56). □

Theorem A1: Assume $R_{m,n}:=R\left(X_{m},Y_{n}\right)$ denotes the FR test statistic as before. Then, the rate for the bias of the $R_{m,n}$ estimator for 0<η≤1, d≥2 is of the form:

(A60) $$\begin{array}{c} |\frac{E\left[R_{m,n}\right]}{m+n}-2pq\int \frac{f_{0}\left(x\right)f_{1}\left(x\right)}{pf_{0}\left(x\right)+qf_{1}\left(x\right)}\;dx|\leq O\left(l^{d}{\left(m+n\right)}^{-\eta /d}\right)+O\left(l^{-d\eta }\right). \end{array}$$

Here, η is the Holder smoothness parameter. A more explicit form for the bound on the RHS is given in (A61) below:

(A61) $$\begin{array}{c} |\frac{E\left[R_{m,n}^{\prime }\left(X_{m},Y_{n}\right)\right]}{m+n}-\int \frac{2pqf_{0}\left(x\right)f_{1}\left(x\right)}{pf_{0}\left(x\right)+qf_{1}\left(x\right)}\;dx|\leq O\left(l^{d}{\left(m+n\right)}^{-\eta /d}\right)\\ \;+O\left(l^{d}{\left(m+n\right)}^{-1/2}\right)+2\;c_{1}\;l^{d-1}{\left(m+n\right)}^{(1/d)-1}+c_{d}\;\;2^{d}\;{\left(m+n\right)}^{-1}\\ -2\;l^{d}{\left(m+n\right)}^{-1}\int \frac{2pqf_{0}\left(x\right)f_{1}\left(x\right)}{p\;f_{0}\left(x\right)+q\;f_{1}\left(x\right)}\;dx+c_{2}\;{\left(m+n\right)}^{-1}l^{d}\\ +O\left(l\right){\left(m+n\right)}^{-1}\sum_{i=1}^{M}l^{d}{\left(a_{i}\right)}^{-1}\int \frac{2f_{0}\left(x\right)f_{1}\left(x\right)}{p\;f_{0}\left(x\right)+q\;f_{1}\left(x\right)}\;dx+O\left(l^{-d\eta }\right)\\ +O\left(l\right)\sum_{i=1}^{M}l^{d/2}\frac{\sqrt{b_{i}}}{a_{i}^{2}}\int \frac{2f_{0}\left(x\right)f_{1}\left(x\right)\left(f_{0}\left(x\right)\sqrt{m}+f_{1}\left(x\right)\sqrt{n}\right)}{{\left(mf_{0}\left(x\right)+nf_{1}\left(x\right)\right)}^{2}}\;dx\\ \;+\sum_{i=1}^{M}2\;l^{-d/2}\frac{\sqrt{b_{i}}}{a_{i}^{2}}\int \frac{f_{0}\left(x\right)f_{1}\left(x\right){\left(\alpha_{i}\beta_{i}\left(ma_{i}f_{0}^{2}\left(x\right)+nb_{i}f_{1}^{2}\left(x\right)\right)\right)}^{1/2}}{{\left(mf_{0}\left(x\right)+nf_{1}\left(x\right)\right)}^{2}\left(m+n\right)}\;dx. \end{array}$$

**Proof.** Assume $M_{m}$ and $N_{n}$ be Poisson variables with mean *m* and *n*, respectively, one independent of another and of $\left\{X_{i}\right\}$ and $\left\{Y_{j}\right\}$. Let also $X_{m}^{\prime }$ and $Y_{n}^{\prime }$ be the Poisson processes $\left\{X_{1},\cdots ,X_{M_{n}}\right\}$ and $\left\{Y_{1},\cdots ,Y_{N_{n}}\right\}$. Set $R_{m,n}^{\prime }:=R_{m,n}\left(X_{m}^{\prime },Y_{n}^{\prime }\right)$. Applying Lemma 1, and (12) cf. [49], we can write

(A62) $$\begin{array}{c} |R_{m,n}^{\prime }-R_{m,n}|\leq K_{d}\left(\right|M_{m}-m|+|N_{n}-n\left|\right). \end{array}$$

Here, $K_{d}$ denotes the largest possible degree of any vertex of the MST graph in $R^{d}$. Moreover, by the matter of Poisson variable fact and using Stirling approximation [51], we have

(A63) $$E\left[|M_{m}-m|\right]=e^{-m}\frac{m^{m+1}}{m!}\leq e^{-m}\frac{m^{m+1}}{\sqrt{2\pi }m^{m+1/2}e^{-m}}=O\left(\;m^{1/2}\right).$$

Similarly, $E\left[|N_{n}-n|\right]=O\left(n^{1/2}\right)$. Therefore, by (A62), one yields

(A64) $$\begin{array}{c} \begin{array}{c} E\left[R_{m,n}\right]=E\left[R_{m,n}-R_{m,n}^{\prime }\right]+E\left[R_{m,n}^{\prime }\right]=O\left({\left(m+n\right)}^{1/2}\right)+E\left[R_{m,n}^{\prime }\right]. \end{array} \end{array}$$

Therefore,

(A65) $$\begin{array}{c} \frac{E[R_{m,n}]}{m+n}=\frac{E\left[R_{m,n}^{\prime }\right]}{m+n}+O\left({\left(m+n\right)}^{-1/2}\right). \end{array}$$

Hence, it will suffice to obtain the rate of convergence of $E\left[R_{m,n}^{\prime }\right]/\left(m+n\right)$ in the RHS of (A65). For this, let $m_{i}$, $n_{i}$ denote the number of Poisson process samples $X_{m}^{\prime }$ and $Y_{n}^{\prime }$ with the FR statistic $R_{m,n}^{\prime }$, falling into partitions $Q_{i}^{\prime }$ with FR statistic $R_{m_{i},n_{i}}^{\prime }$. Then, by virtue of Lemma 4, we can write

$$\begin{array}{c} E\left[R_{m,n}^{\prime }\right]\leq \sum_{i=1}^{M}E\left[R_{m_{i},n_{i}}^{\prime }\right]+2\;c_{1}\;l^{d-1}{\left(m+n\right)}^{1/d}. \end{array}$$

Note that the Binomial RVs $m_{i}$, $n_{i}$ are independent with marginal distributions $m_{i}∼B\left(m,a_{i}l^{-d}\right)$, $n_{i}∼B\left(n,b_{i}l^{-d}\right)$, where $a_{i}$, $b_{i}$ are non-negative constants satisfying, $\forall i,\;a_{i}\leq b_{i}$ and $\sum_{i=1}^{l^{d}}a_{i}l^{-d}=\sum_{i=1}^{l^{d}}b_{i}l^{-d}=1$. Therefore,

(A66) $$E\left[R_{m,n}^{\prime }\right]\leq \sum_{i=1}^{M}E\left[E\left[R_{m_{i},n_{i}}^{\prime }|m_{i},n_{i}\right]\right]+2\;c_{1}\;l^{d-1}{\left(m+n\right)}^{1/d}.$$

Let us first compute the internal expectation given $m_{i}$, $n_{i}$. For this reason, given $m_{i}$, $n_{i}$, let $Z_{1}^{m_{i},n_{i}},Z_{2}^{m_{i},n_{i}},\cdots$ be independent variables with common densities $g_{m_{i},n_{i}}\left(x\right)=\left(m_{i}f_{0}\left(x\right)+n_{i}f_{1}\left(x\right)\right)/\left(m_{i}+n_{i}\right)$, $x\in R^{d}$. Moreover, let $L_{m_{i},n_{i}}$ be an independent Poisson variable with mean $m_{i}+n_{i}$. Denote $F_{m_{i},n_{i}}^{\prime }=\left\{Z_{1}^{m_{i},n_{i}},\cdots ,Z_{L_{m_{i}.n_{i}}}^{m_{i},n_{i}}\right\}$ a non-homogeneous Poisson of rate $m_{i}f_{0}+n_{i}f_{1}$. Let $F_{m_{i},n_{i}}$ be the non-Poisson point process $\left\{Z_{1}^{m_{i},n_{i}},\cdots Z_{m_{i}+n_{i}}^{m_{i},n_{i}}\right\}$. Assign a mark from the set {1,2} to each points of $F_{m_{i},n_{i}}^{\prime }$. Let ${\tilde{X}}_{m_{i}}^{\prime }$ be the sets of points marked 1 with each probability $m_{i}f_{0}\left(x\right)/\left(m_{i}f_{0}\left(x\right)+n_{i}f_{i}\left(x\right)\right)$ and let ${\tilde{Y}}_{n_{i}}^{\prime }$ be the set points with mark 2. Note that owing to the marking theorem [52], ${\tilde{X}}_{m_{i}}^{\prime }$ and ${\tilde{Y}}_{n_{i}}^{\prime }$ are independent Poisson processes with the same distribution as $X_{m_{i}}^{\prime }$ and $Y_{n_{i}}^{\prime }$, respectively. Considering ${\tilde{R}}_{m_{i}.n_{i}}^{\prime }$ as FR statistic over nodes in ${\tilde{X}}_{m_{i}}^{\prime }\cup {\tilde{Y}}_{n_{i}}^{\prime }$ we have

$$\begin{array}{c} E\left[R_{m_{i},n_{i}}^{\prime }|m_{i},n_{i}\right]=E\left[{\tilde{R}}_{m_{i},n_{i}}^{\prime }|m_{i},n_{i}\right]. \end{array}$$

Again using Lemma 1 and analogous arguments in [49] along with the fact that $E\left[|M_{m}+N_{n}-m-n|\right]=O\left({\left(m+n\right)}^{1/2}\right)$, we have

$$\begin{array}{c} \begin{array}{c} E\left[{\tilde{R}}_{m_{i},n_{i}}^{\prime }|m_{i},n_{i}\right]=E\left[E\left[{\tilde{R}}_{m_{i},n_{i}}^{\prime }|F_{m_{i},n_{i}}^{\prime }\right]\right]\\ =E\left[\underset{s<j<m_{i}+n_{i}}{\sum \sum }P_{m_{i},n_{i}}\left(Z_{s}^{m_{i},n_{i}},Z_{j}^{m_{i},n_{i}}\right)1\left\{\left(Z_{s}^{m_{i},n_{i}},Z_{j}^{m_{i},n_{i}}\right)\in F_{m_{i},n_{i}}\right\}\right]+O\left({\left(m_{i}+n_{i}\right)}^{1/2}\right)). \end{array} \end{array}$$

Here,

$$\begin{array}{c} \begin{array}{c} P_{m_{i},n_{i}}\left(x,y\right):=P_{r}\left\{\mathrm{mark}\;x\neq \mathrm{mark}\;y,\left(x,y\right)\in F_{m_{i},n_{i}}^{\prime }\right\}\\ \;=\frac{m_{i}f_{0}\left(x\right)n_{i}f_{1}\left(y\right)+n_{i}f_{1}\left(x\right)m_{i}f_{0}\left(y\right)}{\left(m_{i}f_{0}\left(x\right)+n_{i}f_{1}\left(x\right)\right)\left(m_{i}f_{0}\left(y\right)+n_{i}f_{1}\left(y\right)\right)}. \end{array} \end{array}$$

By owing to Lemma A6, we obtain

(A67) $$\begin{array}{c} \begin{array}{c} \sum_{i=1}^{M}E_{m_{i},n_{i}}E\left[\underset{s<j<m_{i}+n_{i}}{\sum \sum }P_{m_{i},n_{i}}\left(Z_{s}^{m_{i},n_{i}},Z_{j}^{m_{i},n_{i}}\right)1\left\{\left(Z_{s}^{m_{i},n_{i}},Z_{j}^{m_{i},n_{i}}\right)\in F_{m_{i},n_{i}}\right\}\right]+\sum_{i=1}^{M}E_{m_{i},n_{i}}\left[O{\left((m_{i}+n_{i})\right)}^{1/2}\right]\\ =\sum_{i=1}^{M}E_{m_{i},n_{i}}[\left(m_{i}+n_{i}\right)\int g_{m_{i},n_{i}}\left(x,x\right)P_{m_{i},n_{i}}\left(x,x\right)\;dx+(\varsigma_{\eta }\left(l,m_{i},n_{i}\right)+O\left({\left(m_{i}+n_{i}\right)}^{-\eta /d}\right)\\ +O\left({\left(m_{i}+n_{i}\right)}^{-1/d}\right))\left(m_{i}+n_{i}\right)]+\sum_{i=1}^{M}E_{m_{i},n_{i}}\left[O\left({\left(m_{i}+n_{i}\right)}^{1/2}\right)\right], \end{array} \end{array}$$

where

$$\begin{array}{c} \varsigma_{\eta }\left(l,m_{i},n_{i}\right)=\left(O\left(l/(m_{i}+n_{i})\right)-2\;l^{d}/\left(m_{i}+n_{i}\right)\right)\int g_{m_{i},n_{i}}\left(x\right)P_{m_{i},n_{i}}\left(x,x\right)\;dx+O\left(l^{-d\eta }\right). \end{array}$$

The expression in (A67) equals

(A68) $$\begin{array}{c} \sum_{i=1}^{M}\int E_{m_{i},n_{i}}\left[\frac{2m_{i}n_{i}f_{0}\left(x\right)f_{1}\left(x\right)}{m_{i}f_{0}\left(x\right)+n_{i}f_{1}\left(x\right)}\right]\;dx+\sum_{i=1}^{M}E_{m_{i},n_{i}}\left[\left(m_{i}+n_{i}\right)\;\varsigma_{\eta }\left(l,m_{i},n_{i}\right)\right]\\ \;\;+O\left(l^{d}{\left(m+n\right)}^{1-\eta /d}\right)+O\left(l^{d}{\left(m+n\right)}^{1/2}\right). \end{array}$$

Because of Jensen inequality for concave function:

$$\begin{array}{c} \begin{array}{c} \sum_{i=1}^{M}E_{m_{i},n_{i}}\left[O\left({\left(m_{i}+n_{i}\right)}^{1/2}\right)\right]=\sum_{i=1}^{M}O{\left(E\left[m_{i}\right]+E\left[n_{i}\right]\right)}^{1/2}\\ \;\;=\sum_{i=1}^{M}O{\left(ma_{i}l^{-d}+nb_{i}l^{-d}\right)}^{1/2}=O\left(l^{d}{\left(m+n\right)}^{1/2}\right). \end{array} \end{array}$$

In addition, similarly since η<d, we have

(A69) $$\begin{array}{c} \sum_{i=1}^{M}E_{m_{i},n_{i}}\left[O\left({\left(m_{i}+n_{i}\right)}^{1-\eta /d}\right)\right]=O\left(l^{d}{\left(m+n\right)}^{1-\eta /d}\right), \end{array}$$

and, for d≥2, one yields

(A70) $$\begin{array}{c} \begin{array}{c} \sum_{i=1}^{M}E_{m_{i},n_{i}}\left[O\left({\left(m_{i}+n_{i}\right)}^{1-1/d}\right)\right]=O\left(l^{d}{\left(m+n\right)}^{1-1/d}\right)=O\left(l^{d}{\left(m+n\right)}^{1/2}\right). \end{array} \end{array}$$

Next, we state the following lemma (Lemma 1 from [30,31]), which will be used in the sequel: **Lemma** **A13.**
*Let k(x) be a continuously differential function of x∈R which is convex and monotone decreasing over x≥0. Set $k^{\prime }\left(x\right)=\frac{dk(x)}{dx}$. Then, for any $x_{0}>0$, we have*

(A71) $$\begin{array}{c} k\left(x_{0}\right)+\frac{k(x_{0})}{x_{0}}|x-x_{0}|\geq k\left(x\right)\geq k\left(x_{0}\right)-k^{\prime }\left(x_{0}\right)\left|x-x_{0}\right|. \end{array}$$

Next, continuing the proof of (A60), we attend to find an upper bound for

(A72) $$\begin{array}{c} E_{m_{i},n_{i}}\left[\frac{m_{i}n_{i}}{m_{i}f_{0}\left(x\right)+n_{i}f_{1}\left(x\right)}\right]. \end{array}$$

In order to pursue this aim, in Lemma A13, consider $k\left(x\right)=\frac{1}{x}$ and $x_{0}=E_{m_{i},n_{i}}\left[m_{i}f_{0}\left(x\right)+n_{i}f_{1}\left(x\right)\right]$, therefore as the function k(x) is decreasing and convex, one can write

(A73) $$\begin{array}{c} \frac{1}{m_{i}f_{0}\left(x\right)+n_{i}f_{1}\left(x\right)}\leq \frac{1}{E_{m_{i},n_{i}}\left[m_{i}f_{0}\left(x\right)+n_{i}f_{1}\left(x\right)\right]}+\frac{\left|m_{i}f_{0}\left(x\right)+n_{i}f_{1}\left(x\right)-E_{m_{i},n_{i}}\left[m_{i}f_{0}\left(x\right)+n_{i}f_{1}\left(x\right)\right]\right|}{E_{m_{i},n_{i}}^{2}\left[m_{i}f_{0}\left(x\right)+n_{i}f_{1}\left(x\right)\right]}. \end{array}$$

Using the Hölder inequality implies the following inequality:

(A74) $$\begin{array}{c} \begin{array}{c} E_{m_{i},n_{i}}\left[\frac{m_{i}n_{i}}{m_{i}f_{0}\left(x\right)+n_{i}f_{1}\left(x\right)}\right]\leq \frac{E_{m_{i},n_{i}}\left[m_{i}n_{i}\right]}{E_{m_{i},n_{i}}\left[m_{i}f_{0}\left(x\right)+n_{i}f_{1}\left(x\right)\right]}\\ +\frac{{\left(E_{m_{i},n_{i}}\left[m_{i}^{2}n_{i}^{2}\right]\right)}^{1/2}}{E_{m_{i},n_{i}}^{2}\left[m_{i}f_{0}\left(x\right)+n_{i}f_{1}\left(x\right)\right]}\times {\left(E_{m_{i},n_{i}}{\left[m_{i}f_{0}\left(x\right)+n_{i}f_{1}\left(x\right)-E_{m_{i},n_{i}}\left[m_{i}f_{0}\left(x\right)+n_{i}f_{1}\left(x\right)\right]\right]}^{2}\right)}^{1/2}. \end{array} \end{array}$$

As random variables $m_{i}$, $n_{i}$ are independent, and because of $V\left[m_{i}\right]\leq ma_{i}l^{-d}$, $V\left[n_{i}\right]\leq nb_{i}l^{-d}$, we can claim that the RHS of (A74) becomes less than and equal to

(A75) $$\begin{array}{c} \begin{array}{c} \frac{mna_{i}b_{i}l^{-2d}}{ma_{i}l^{-d}f_{0}\left(x\right)+nb_{i}l^{-d}f_{1}\left(x\right)}+\frac{{\left(\alpha_{i}\beta_{i}\left(ma_{i}l^{-d}f_{0}^{2}\left(x\right)+nb_{i}l^{-d}f_{1}^{2}\left(x\right)\right)\right)}^{1/2}}{{\left(ma_{i}f_{0}\left(x\right)+nb_{i}f_{1}\left(x\right)\right)}^{2}}, \end{array} \end{array}$$

where

$$\begin{array}{c} \begin{array}{c} \alpha_{i}=ma_{i}l^{d}\;\left(1-a_{i}l^{-d}\right)+m^{2}a_{i}^{2},\\ \beta_{i}=nb_{i}l^{d}\;\left(1-b_{i}l^{-d}\right)+n^{2}b_{i}^{2}. \end{array} \end{array}$$

Going back to (A66), we have

(A76) $$\begin{array}{c} E\left[{R^{\prime }}_{m,n}\left(X_{m},Y_{n}\right)\right]\leq \sum_{i=1}^{M}\;a_{i}b_{i}l^{-d}\int \frac{2\;mnf_{0}\left(x\right)f_{1}\left(x\right)}{ma_{i}f_{0}\left(x\right)+nb_{i}f_{1}\left(x\right)}\;dx\\ +\sum_{i=1}^{M}2\int \frac{f_{0}\left(x\right)f_{1}\left(x\right){\left(\alpha_{i}\beta_{i}\left(ma_{i}l^{-d}f_{0}^{2}\left(x\right)+nb_{i}l^{-d}f_{1}^{2}\left(x\right)\right)\right)}^{1/2}}{{\left(ma_{i}f_{0}\left(x\right)+nb_{i}f_{1}\left(x\right)\right)}^{2}}\;dx\\ \;+\sum_{i=1}^{M}E_{m_{i},n_{i}}\left[\left(m_{i}+n_{i}\right)\;\varsigma_{\eta }\left(l,m_{i},n_{i}\right)\right]+O\left(l^{d}{\left(m+n\right)}^{1-\eta /d}\right)\\ \;+O\left(l^{d}{\left(m+n\right)}^{1/2}\right)+2c_{1}\;l^{d-1}{\left(m+n\right)}^{1/d}. \end{array}$$

Finally, owing to $a_{i}\leq b_{i}$ and $\sum_{i=1}^{M}b_{i}l^{-d}=1$, when $\frac{m}{m+n}\to p$, we have

(A77) $$\begin{array}{c} \frac{E\left[{R^{\prime }}_{m,n}\left(X_{m},Y_{n}\right)\right]}{m+n}\leq \int \frac{2\;pqf_{0}\left(x\right)f_{1}\left(x\right)}{pf_{0}\left(x\right)+qf_{1}\left(x\right)}\;dx\\ \;+\sum_{i=1}^{M}2\int \frac{f_{0}\left(x\right)f_{1}\left(x\right){\left(\alpha_{i}\beta_{i}\left(ma_{i}l^{-d}f_{0}^{2}\left(x\right)+nb_{i}l^{-d}f_{1}^{2}\left(x\right)\right)\right)}^{1/2}}{{\left(ma_{i}f_{0}\left(x\right)+nb_{i}f_{1}\left(x\right)\right)}^{2}\left(m+n\right)}\;dx\\ \;+\frac{1}{m+n}\sum_{i=1}^{M}E_{m_{i},n_{i}}\left[\left(m_{i}+n_{i}\right)\;\varsigma_{\eta }\left(l,m_{i},n_{i}\right)\right]+O\left(l^{d}{\left(m+n\right)}^{-\eta /d}\right)\\ \;+O\left(l^{d}{\left(m+n\right)}^{-1/2}\right)+2c_{1}\;l^{d-1}\;{\left(m+n\right)}^{(1/d)-1}. \end{array}$$

Passing to Definition 2, ${\mathrm{MST}}^{*}$, and Lemma A2, a similar discussion as above, consider the Poisson processes samples and the FR statistic under the union of samples, denoted by ${R^{\prime }}_{m,n}^{*}$, and superadditivity of dual $R_{m,n}^{*}$, we have

(A78) $$\begin{array}{c} \begin{array}{c} E\left[{R^{\prime }}_{m,n}^{*}\left(X_{m},Y_{n}\right)\right]\geq \sum_{i=1}^{M}E\left[{R^{\prime }}_{m_{i},n_{i}}^{*}\left(\left(X_{m},Y_{n}\right)\cap Q_{i}\right)\right]-\;c_{2}\;l^{d}\\ \;=\sum_{i=1}^{M}E_{m_{i},n_{i}}\left[E\left[{R^{\prime }}_{m_{i},n_{i}}^{*}\left(\left(X_{m},Y_{n}\right)\cap Q_{i}\right)|m_{i},n_{i}\right]\right]-c_{2}\;l^{d}\\ \;\geq \sum_{i=1}^{M}E_{m_{i},n_{i}}\left[E\left[{R^{\prime }}_{m_{i},n_{i}}\left(\left(X_{m},Y_{n}\right)\cap Q_{i}\right)|m_{i},n_{i}\right]\right]-c_{2}\;l^{d}, \end{array} \end{array}$$

the last line is derived from Lemma A2, (ii), inequality (A8). Owing to the Lemma A6, (A69), and (A70), one obtains

(A79) $$\begin{array}{c} E\left[{R^{\prime }}_{m,n}^{*}\left(X_{m},Y_{n}\right)\right]\geq \sum_{i=1}^{M}\int E_{m_{i},n_{i}}\left[\frac{2m_{i}n_{i}f_{0}\left(x\right)f_{1}\left(x\right)}{m_{i}f_{0}\left(x\right)+n_{i}f_{1}\left(x\right)}\right]\;dx\\ -\sum_{i=1}^{M}E_{m_{i},n_{i}}\left[\left(m_{i}+n_{i}\right)\;\varsigma_{\eta }\left(l,m_{i},n_{i}\right)\right]-O\left(l^{d}{\left(m+n\right)}^{1-\eta /d}\right)-O\left(l^{d}{\left(m+n\right)}^{1/2}\right)-c_{2}\;l^{d}. \end{array}$$

Furthermore, by using the Jenson’s inequality, we get

$$\begin{array}{c} \begin{array}{c} E_{m_{i},n_{i}}\left[\frac{m_{i}n_{i}}{m_{i}f_{0}\left(x\right)+n_{i}f_{1}\left(x\right)}\right]\geq \frac{E\left[m_{i}\right]E\left[n_{i}\right]}{E\left[m_{i}\right]f_{0}\left(x\right)+E\left[n_{i}\right]f_{1}\left(x\right)}=\frac{l^{-d}\left(ma_{i}nb_{i}\right)}{ma_{i}f_{0}\left(x\right)+nb_{i}f_{1}\left(x\right)}. \end{array} \end{array}$$

Therefore, since $a_{i}\leq b_{i}$, we can write

(A80) $$\begin{array}{c} E_{m_{i},n_{i}}\left[\frac{m_{i}n_{i}}{m_{i}f_{0}\left(x\right)+n_{i}f_{1}\left(x\right)}\right]\geq \frac{l^{-d}mn\;a_{i}b_{i}}{b_{i}\left(mf_{0}\left(x\right)+nf_{1}\left(x\right)\right)}=\frac{l^{-d}mn\;a_{i}}{\left(mf_{0}\left(x\right)+nf_{1}\left(x\right)\right)}. \end{array}$$

Consequently, the RHS of (A79) becomes greater than or equal to

(A81) $$\begin{array}{c} \sum_{i=1}^{M}a_{i}\;l^{-d}\int \frac{2mnf_{0}\left(x\right)f_{1}\left(x\right)}{mf_{0}\left(x\right)+nf_{1}\left(x\right)}\;dx\\ \;-\sum_{i=1}^{M}E_{m_{i},n_{i}}\left[\left(m_{i}+n_{i}\right)\;\varsigma_{\eta }\left(l,m_{i},n_{i}\right)\right]-O\left(l^{d}{\left(m+n\right)}^{1-\eta /d}\right)-O\left(l^{d}{\left(m+n\right)}^{1/2}\right)-c_{2}\;l^{d}. \end{array}$$

Finally, since $\sum_{i=1}^{M}a_{i}l^{-d}=1$ and $\frac{m}{m+n}\to p$, we have

(A82) $$\begin{array}{c} \frac{E\left[{R^{\prime }}_{m,n}^{*}\left(X_{m},Y_{n}\right)\right]}{m+n}\geq \int \frac{2pqf_{0}\left(x\right)f_{1}\left(x\right)}{pf_{0}\left(x\right)+qf_{1}\left(x\right)}\;dx-{\left(m+n\right)}^{-1}\sum_{i=1}^{M}E_{m_{i},n_{i}}\left[\left(m_{i}+n_{i}\right)\;\varsigma \left(l,m_{i},n_{i}\right)\right]\\ \;-O\left(l^{d}{\left(m+n\right)}^{-\eta /d}\right)-O\left(l^{d}{\left(m+n\right)}^{-1/2}\right)-c_{2}\;l^{d}{\left(m+n\right)}^{-1}. \end{array}$$

By definition of the dual $R_{m,n}^{*}$ and (i) in Lemma A2,

(A83) $$\begin{array}{c} \frac{E\left[R_{m,n}^{\prime }\left(X_{m},Y_{n}\right)\right]}{m+n}+\frac{c_{d}\;2^{d}}{m+n}\geq \frac{E\left[{R^{\prime }}_{m,n}^{*}\left(X_{m},Y_{n}\right)\right]}{m+n}, \end{array}$$

we can imply

(A84) $$\begin{array}{c} \begin{array}{c} \frac{E\left[{R^{\prime }}_{m,n}\left(X_{m},Y_{n}\right)\right]}{m+n}\geq \int \frac{2pqf_{0}\left(x\right)f_{1}\left(x\right)}{pf_{0}\left(x\right)+qf_{1}\left(x\right)}\;dx-{\left(m+n\right)}^{-1}\sum_{i=1}^{M}E_{m_{i},n_{i}}\left[\left(m_{i}+n_{i}\right)\;\varsigma_{\eta }\left(l,m_{i},n_{i}\right)\right]\\ \;-O\left(l^{d}{\left(m+n\right)}^{-\eta /d}\right)-O\left(l^{d}{\left(m+n\right)}^{-1/2}\right)-c_{2}\;l^{d}{\left(m+n\right)}^{-1}-c_{d}\;2^{d}\;{\left(m+n\right)}^{-1}. \end{array} \end{array}$$

The combination of two lower and upper bounds (A84) and (A77) yields the following result

(A85) $$\begin{array}{c} \left|\frac{E\left[R_{m,n}^{\prime }\left(X_{m},Y_{n}\right)\right]}{m+n}-\int \frac{2pqf_{0}\left(x\right)f_{1}\left(x\right)}{pf_{0}\left(x\right)+qf_{1}\left(x\right)}\;dx\right|\\ \leq O\left(l^{d}{\left(m+n\right)}^{-\eta /d}\right)+O\left(l^{d}{\left(m+n\right)}^{-1/2}\right)+2\;c_{1}\;l^{d-1}\;{\left(m+n\right)}^{(1/d)-1}\\ +c_{d}\;2^{d}\;{\left(m+n\right)}^{-1}+c_{2}\;{\left(m+n\right)}^{-1}\;l^{d}+\frac{1}{m+n}\sum_{i=1}^{M}E_{m_{i},n_{i}}\left[\left(m_{i}+n_{i}\right)\;\varsigma_{\eta }\left(l,m_{i},n_{i}\right)\right]\\ +\sum_{i=1}^{M}2\int \frac{f_{0}\left(x\right)f_{1}\left(x\right){\left(\alpha_{i}\beta_{i}\left(ma_{i}l^{-d}f_{0}^{2}\left(x\right)+nb_{i}l^{-d}f_{1}^{2}\left(x\right)\right)\right)}^{1/2}}{{\left(ma_{i}f_{0}\left(x\right)+nb_{i}f_{1}\left(x\right)\right)}^{2}\left(m+n\right)}\;dx. \end{array}$$

Recall $\varsigma_{\eta }\left(l,m_{i},n_{i}\right)$, then we obtain

(A86) $$\begin{array}{c} \begin{array}{c} \sum_{i=1}^{M}E_{m_{i},n_{i}}\left[\left(m_{i}+n_{i}\right)\;\varsigma_{\eta }\left(l,m_{i},n_{i}\right)\right]=\sum_{i=1}^{M}O\left(l\right)\int E\left[\frac{2m_{i}n_{i}f_{0}\left(x\right)f_{1}\left(x\right)}{\left(m_{i}+n_{i}\right)\left(m_{i}f_{0}\left(x\right)+n_{i}f_{1}\left(x\right)\right)}\right]\;dx\\ \;\;-2\;l^{d}\sum_{i=1}^{M}\int E\left[\frac{2m_{i}n_{i}f_{0}\left(x\right)f_{1}\left(x\right)}{\left(m_{i}+n_{i}\right)\left(m_{i}f_{0}\left(x\right)+n_{i}f_{1}\left(x\right)\right)}\right]\;dx+O\left(l^{-\eta }\right)\sum_{i=1}^{M}E_{m_{i},n_{i}}\left[m_{i}+n_{i}\right]. \end{array} \end{array}$$

In addition, we have

(A87) $$\begin{array}{c} \begin{array}{c} E_{m_{i},n_{i}}\left[\frac{2m_{i}n_{i}f_{0}\left(x\right)f_{1}\left(x\right)}{\left(m_{i}+n_{i}\right)\left(m_{i}f_{0}\left(x\right)+n_{i}f_{1}\left(x\right)\right)}\right]\geq \frac{1}{m+n}E_{m_{i},n_{i}}\left[\frac{2m_{i}n_{i}f_{0}\left(x\right)f_{1}\left(x\right)}{\left(m_{i}f_{0}\left(x\right)+n_{i}f_{1}\left(x\right)\right)}\right]. \end{array} \end{array}$$

This implies

(A88) $$\begin{array}{c} \begin{array}{c} \sum_{i=1}^{M}\int E\left[\frac{2m_{i}n_{i}f_{0}\left(x\right)f_{1}\left(x\right)}{\left(m_{i}+n_{i}\right)\left(m_{i}f_{0}\left(x\right)+n_{i}f_{1}\left(x\right)\right)}\right]\;dx\geq \int \frac{2pqf_{0}\left(x\right)f_{1}\left(x\right)}{pf_{0}\left(x\right)+qf_{1}\left(x\right)}\;dx. \end{array} \end{array}$$

Note that the above inequality is derived from (A80) and $\frac{m}{m+n}\to p$. Furthermore,

(A89) $$\begin{array}{c} \frac{1}{m+n}\sum_{i=1}^{M}O\left(l\right)\int E_{m_{i},n_{i}}\left[\frac{2m_{i}n_{i}f_{0}\left(x\right)f_{1}\left(x\right)}{\left(m_{i}+n_{i}\right)\left(m_{i}f_{0}\left(x\right)+n_{i}f_{1}\left(x\right)\right)}\right]\;dx\\ \;\;\leq \sum_{i=1}^{M}O\left(l\right)\int E_{m_{i},n_{i}}\left[\frac{2m_{i}n_{i}f_{0}\left(x\right)f_{1}\left(x\right)}{{\left(m_{i}+n_{i}\right)}^{2}\left(m_{i}f_{0}\left(x\right)+n_{i}f_{1}\left(x\right)\right)}\right]\;dx\\ \;\;\leq \sum_{i=1}^{M}O\left(l\right)\int E_{m_{i},n_{i}}\left[\frac{2f_{0}\left(x\right)f_{1}\left(x\right)}{\left(m_{i}f_{0}\left(x\right)+n_{i}f_{1}\left(x\right)\right)}\right]\;dx. \end{array}$$

The last line holds because of $m_{i}n_{i}\leq {\left(m_{i}+n_{i}\right)}^{2}$. Going back to (A73), we can give an upper bound for the RHS of above inequality as

$$\begin{array}{c} \begin{array}{c} E_{m_{i},n_{i}}\left[{\left(m_{i}f_{0}\left(x\right)+n_{i}f_{1}\left(x\right)\right)}^{-1}\right]\leq {\left(ma_{i}l^{-d}f_{0}\left(x\right)+nb_{i}l^{-d}f_{1}\left(x\right)\right)}^{-1}\\ \;+(E_{m_{i},n_{i}}|m_{i}f_{0}\left(x\right)+n_{i}f_{1}\left(x\right)-\left(E\left[m_{i}\right]f_{0}\left(x\right)+E\left[n_{i}\right]f_{1}\left(x\right)|\right)/{\left(ma_{i}l^{-d}f_{0}\left(x\right)+nb_{i}l^{-d}f_{1}\left(x\right)\right)}^{2}. \end{array} \end{array}$$

Note that we have assumed $a_{i}\leq b_{i}$ and by using Hölder inequality we write

(A90) $$\begin{array}{c} \begin{array}{c} E_{m_{i},n_{i}}\left[{\left(m_{i}f_{0}\left(x\right)+n_{i}f_{1}\left(x\right)\right)}^{-1}\right]\leq l^{d}{\left(a_{i}\right)}^{-1}{\left(mf_{0}\left(x\right)+nf_{1}\left(x\right)\right)}^{-1}\\ \;+\left(f_{0}\left(x\right)\sqrt{V(m_{i})}+f_{1}\left(x\right)\sqrt{V(n_{i})}\right)/\left(a_{i}^{2}l^{-d}{\left(mf_{0}\left(x\right)+nf_{1}\left(x\right)\right)}^{2}\right)\leq l^{d}{\left(a_{i}\right)}^{-1}{\left(mf_{0}\left(x\right)+nf_{1}\left(x\right)\right)}^{-1}\\ \;+\;l^{-d/2}\sqrt{b_{i}}\left(f_{0}\left(x\right)\sqrt{m}+f_{1}\left(x\right)\sqrt{n}\right)/\left(a_{i}^{2}l^{-d}{\left(mf_{0}\left(x\right)+nf_{1}\left(x\right)\right)}^{2}\right). \end{array} \end{array}$$

As result, we have

(A91) $$\begin{array}{c} \sum_{i=1}^{M}O\left(l\right)\int E_{m_{i},n_{i}}\left[\frac{2f_{0}\left(x\right)f_{1}\left(x\right)}{\left(m_{i}f_{0}\left(x\right)+n_{i}f_{1}\left(x\right)\right)}\right]\;dx\\ \;\;\leq \sum_{i=1}^{M}O\left(l\right)\int l^{d}{\left(a_{i}\right)}^{-1}\frac{2f_{0}\left(x\right)f_{1}\left(x\right)}{mf_{0}\left(x\right)+nf_{1}\left(x\right)}\;dx\\ \;\;\;+\sum_{i=1}^{M}O\left(l\right)\int l^{-d/2}\sqrt{b_{i}}\;\frac{2f_{0}\left(x\right)f_{1}\left(x\right)\left(f_{0}\left(x\right)\sqrt{m}+f_{1}\left(x\right)\sqrt{n}\right)}{a_{i}^{2}l^{-d}{\left(mf_{0}\left(x\right)+nf_{1}\left(x\right)\right)}^{2}}\;dx. \end{array}$$

As a consequence, owing to (A85), for 0<η≤1, d≥2, which implies η≤d−1, we can derive (A61). Thus, the proof can be concluded by giving the summarized bound in (A60). □

Lemma A8: For h=1,2,⋯, let $\delta_{m,n}^{h}$ be the function $c\;h^{d-1}{\left(m+n\right)}^{1/d}$. Then, for ϵ>0, we have

(A92) $$P\left(R_{m,n}\left(X_{m},Y_{n}\right)\leq \sum_{i=1}^{h^{d}}R_{m_{i},n_{i}}\left(X_{m_{i}},Y_{n_{i}}\right)+2\epsilon \right)\geq \frac{\epsilon -\delta_{m,n}^{h}}{\epsilon }.$$

Note that in case $\epsilon \leq \delta_{m,n}^{h}$ the above claimed inequality is trivial.

**Proof.** Consider the cardinality of the set of all edges of $\mathrm{MST}\left(\overset{h^{d}}{\underset{i=1}{⋃}}Q_{i}\right)$ which intersect two different subcubes $Q_{i}$ and $Q_{j}$, |D|. Using the Markov inequality, we can write

$$\begin{array}{c} P\left(|D|\geq \epsilon \right)\leq \frac{E(|D|)}{\epsilon }, \end{array}$$

where ϵ>0. Since $E|D|\leq c\;h^{d-1}{\left(m+n\right)}^{1/d}:=\delta_{m,n}^{h}$, therefore for $\epsilon >\delta_{m,n}^{h}$ and h=1,2,⋯:

$$\begin{array}{c} P\left(|D|\geq \epsilon \right)\leq \frac{\delta_{m,n}^{h}}{\epsilon }. \end{array}$$

In addition, if $Q_{i}$, $i=1,\cdots h^{d}$ is a partition of ${\left[0,1\right]}^{d}$ into congruent subcubes of edge length 1/h, then

(A93) $$\begin{array}{c} P\left(\sum_{i=1}^{h^{d}}R_{m_{i},n_{i}}\left(X_{m},Y_{n}\cap Q_{i}\right)+2\left|D\right|\geq \sum_{i=1}^{h^{d}}R_{m_{i},n_{i}}\left(X_{m},Y_{n}\cap Q_{i}\right)+2\epsilon \right)\leq \frac{\delta_{m,n}^{h}}{\epsilon }. \end{array}$$

This implies

(A94) $$\begin{array}{c} P\left(\sum_{i=1}^{h^{d}}R_{m_{i},n_{i}}\left(X_{m},Y_{n}\cap Q_{i}\right)+2\left|D\right|\leq \sum_{i=1}^{h^{d}}R_{m_{i},n_{i}}\left(X_{m},Y_{n}\cap Q_{i}\right)+2\epsilon \right)\geq 1-\frac{\delta_{m,n}^{h}}{\epsilon }. \end{array}$$

By subadditivity (A6), we can write

$$\begin{array}{c} R_{m,n}\left(X_{m},Y_{n}\right)\leq \sum_{i=1}^{h^{d}}R_{m_{i},n_{i}}\left(X_{m},Y_{n}\cap Q_{i}\right)+2\left|D\right|, \end{array}$$

and this along with (A94) establishes (A92). □

Lemma A9: (Growth bounds for $R_{m,n}$) Let $R_{m,n}$ be the FR statistic. Then, for given non-negative ϵ, such that $\epsilon \geq h^{2}\;\delta_{m,n}^{h}$, with at least probability $g\left(\epsilon \right):=1-\frac{h\;\delta_{m,n}^{h}}{\epsilon }$, h=2,3,⋯, we have

(A95) $$\begin{array}{c} R_{m,n}\left(X_{m},Y_{n}\right)\leq c_{\epsilon ,h}^{\prime \prime }\;{\left(\#X_{m}\;\#Y_{n}\right)}^{1-1/d}. \end{array}$$

Here, $c_{\epsilon ,h}^{\prime \prime }=O\left(\frac{\epsilon }{h^{d-1}-1}\right)$ depending only on ϵ, *h*. Note that, for $\epsilon <h^{2}\;\delta_{m,n}^{h}$, the claim is trivial.

**Proof.** Without loss of generality, consider the unit cube ${\left[0,1\right]}^{d}$. For given *h*, if $Q_{i}$, $i=1,\cdots h^{d}$ is a partition of ${\left[0,1\right]}^{d}$ into congruent subcubes of edge length 1/h, then, by Lemma A8, we have

(A96) $$P\left(R_{m,n}\left(X_{m},Y_{n}\right)\leq \sum_{i=1}^{h^{d}}R_{m_{i},n_{i}}\left(X_{m_{i}},Y_{n_{i}}\right)+2\epsilon \right)\geq \frac{\epsilon -\delta_{m,n}^{h}}{\epsilon }.$$

We apply the induction methodology on $\#X_{m}$ and $\#Y_{n}$. Set $c:=\underset{x,y\in {\left[0,1\right]}^{d}}{\sup}R_{m,n}\left(\left\{x,y\right\}\right)$ which is finite according to assumption. Moreover, set $c_{2}:=\frac{2\epsilon }{h^{d-1}-1}$ and $c_{1}:=c+d\;h^{d-1}c_{2}$. Therefore, it is sufficient to show that for all $\left(X_{m},Y_{n}\right)\in {\left[0,1\right]}^{d}$ with at least probability g(ϵ)

(A97) $$\begin{array}{c} R_{m,n}\left(X_{m},Y_{n}\right)\leq c_{1}{\left(\#X_{m}\;\#Y_{n}\right)}^{(d-1)/d}. \end{array}$$

Alternatively, as for the induction hypothesis, we assume the stronger bound

(A98) $$\begin{array}{c} R_{m,n}\left(X_{m},Y_{n}\right)\leq c_{1}{\left(\#X_{m}\;\#Y_{n}\right)}^{(d-1)/d}-c_{2} \end{array}$$

holds whenever $\#X_{m}<m$ and $\#Y_{n}<n$ with at least probability g(ϵ). Note that d≥2, ϵ>0 and $c_{1}$, $c_{2}$ both depend on ϵ, *h*. Hence,

$$\begin{array}{c} c_{1}-c_{2}=c+c_{2}\left(d\;h^{d-1}-1\right)\geq c+c_{2}\left(h^{d-1}-1\right)=c+2\epsilon \geq c, \end{array}$$

which implies $P\left(R_{m,n}\leq c_{1}-c_{2}\right)\geq P\left(R_{m,n}\leq c\right)$. In addition, we know that $P\left(R_{m,n}\leq c\right)=1\geq g\left(\epsilon \right)$; therefore, the induction hypothesis holds particularly $\#X_{m}=1$ and $\#Y_{n}=1$. Now, consider the partition $Q_{i}$ of ${\left[0,1\right]}^{d}$; therefore, for all $1\leq i\leq h^{d}$, we have $m_{i}:=\#\left(X_{m}\cap Q_{i}\right)<m$ and $n_{i}:=\#\left(Y_{n}\cap Q_{i}\right)<n$ and thus, by induction hypothesis, one yields with at least probability g(ϵ)

(A99) $$\begin{array}{c} R_{m_{i},n_{i}}\left(X_{m},Y_{n}\cap Q_{i}\right)\leq c_{1}\;{\left(m_{i}\;n_{i}\right)}^{1-1/d}-c_{2}. \end{array}$$

Set B the event $\left\{\mathrm{all}\;i\;:\;R_{m_{i},n_{i}}\leq c_{1}\;{\left(m_{i}\;n_{i}\right)}^{1-1/d}-c_{2}\right\}$ and $B_{i}$ stands with the event $\left\{R_{m_{i},n_{i}}\leq c_{1}\;{\left(m_{i}\;n_{i}\right)}^{1-1/d}-c_{2}\right\}$. From (A96) and since $Q_{i}$’s are partitions, which implies

$$\begin{array}{c} \begin{array}{c} P\left(B\right)={\left(P(B_{i})\right)}^{h^{d}}\leq P\left(B_{i}\right),\;\;P\left(B^{c}\right)=P\left(\overset{l^{d}}{\underset{i=1}{⋃}}B_{i}^{c}\right)\leq \sum_{i=1}^{h^{d}}P\left(B_{i}^{c}\right)\leq h^{d}\left(1-g(\epsilon )\right),\;\;\\ \;\;\mathrm{and}\;\;\;P\left(B\right)=\prod_{i=1}^{h^{d}}P\left(B_{i}\right)\geq {\left(g(\epsilon )\right)}^{h^{d}}, \end{array} \end{array}$$

we thus obtain

$$\begin{array}{c} \begin{array}{c} \frac{\epsilon -\delta_{m,n}^{h}}{\epsilon }\leq P\left(R_{m,n}\leq \sum_{i=1}^{h^{d}}R_{m_{i},n_{i}}\left(X_{m_{i}},Y_{n_{i}}\right)+2\epsilon |B\right)P\left(B\right)+P\left(R_{m,n}\leq \sum_{i=1}^{h^{d}}R_{m_{i},n_{i}}\left(X_{m_{i}},Y_{n_{i}}\right)+2\epsilon |B^{c}\right)P\left(B^{c}\right)\\ \leq P\left(R_{m,n}\leq \sum_{i=1}^{l^{d}}R_{m_{i},n_{i}}\left(X_{m_{i}},Y_{n_{i}}\right)+2\epsilon |B\right)P\left(B\right)+P\left(B^{c}\right). \end{array} \end{array}$$

Equivalently,

$$\begin{array}{c} \begin{array}{c} P\left(R_{m,n}\leq \sum_{i=1}^{h^{d}}R_{m_{i},n_{i}}\left(X_{m_{i}},Y_{n_{i}}\right)+2\epsilon |B\right)\geq \left(1-\frac{\delta_{m,n}^{h}}{\epsilon }-1+P\left(B\right)\right)/P\left(B\right)=1-\frac{\delta_{m,n}^{h}}{\epsilon \;P(B)}. \end{array} \end{array}$$

In fact, in this stage, we want to show that

$$\begin{array}{c} 1-\frac{\delta_{m,n}^{h}}{\epsilon \;P(B)}\geq g\left(\epsilon \right)\;\;\;\mathrm{or}\;\;\;P\left(B\right)\geq \frac{\delta_{m,n}^{h}}{\epsilon \;(1-g(\epsilon ))}. \end{array}$$

Since $P\left(B\right)\geq {\left(g(\epsilon )\right)}^{h^{d}}$, therefore it is sufficient to derive that ${\left(g(\epsilon )\right)}^{h^{d}}\geq \frac{\delta_{m,n}^{h}}{\epsilon \;(1-g(\epsilon ))}$. Indeed, for given $g\left(\epsilon \right)=\left(\frac{\epsilon -h\;\delta_{m,n}^{h}}{\epsilon }\right)$, we have $g\left(\epsilon \right)\leq \frac{\epsilon -\delta_{m,n}^{h}}{\epsilon }$ hence $\frac{\delta_{m,n}^{h}}{\epsilon \;(1-g(\epsilon ))}=\frac{1}{h}\leq 1$. Furthermore, we know $\frac{1}{h}\leq 1-\frac{1}{h^{\left(1/h^{d}\right)}}$ and since $\epsilon \geq h^{2}\;\delta_{m,n}^{h}$ this implies $\frac{h\;\delta_{m,n}^{h}}{\epsilon }\leq \frac{1}{h}$ and consequently

$$\frac{h\;\delta_{m,n}^{h}}{\epsilon }\leq 1-\frac{1}{h^{h^{-d}}}$$

or

$$g{\left(\epsilon \right)}^{h^{d}}={\left(\frac{\epsilon -h\;\delta_{m,n}^{h}}{\epsilon }\right)}^{h^{d}}\geq \frac{1}{h}=\frac{\delta_{m,n}^{h}}{\epsilon \;(1-g(\epsilon ))}.$$

This implies the fact that for $\epsilon \geq h^{2}\delta_{m,n}^{h}$

$$\begin{array}{c} \begin{array}{c} P\left(R_{m,n}\leq \sum_{i=1}^{h^{d}}\left(c_{1}{\left(m_{i}n_{i}\right)}^{1-1/d}-c_{2}\right)+2\epsilon \right)\geq g\left(\epsilon \right),\;\;\;\mathrm{where}\;\;\;g\left(\epsilon \right)=\frac{\epsilon -h\;\delta_{m,n}^{h}}{\epsilon }. \end{array} \end{array}$$

Now, let $\gamma :=\#\{i:m_{i},n_{i}>0\}$ and using Hölder inequality gives

(A100) $$P\left(R_{m,n}\left(X_{m},Y_{n}\right)\leq c_{1}\gamma^{1/d}{\left(m\;n\right)}^{1-1/d}-\gamma c_{2}+c_{2}\;\left(h^{d-1}-1\right)\right)\geq g\left(\epsilon \right).$$

Next, we just need to show that $c_{1}\gamma^{1/d}{\left(m\;n\right)}^{1-1/d}-\gamma c_{2}+c_{2}\;\left(h^{d-1}-1\right)$ in (A100) is less than or equal to $c_{1}{\left(m\;n\right)}^{1-1/d}-c_{2}$, which is equivalent to show

$$\begin{array}{c} c_{2}\left(h^{d-1}-\gamma \right)\leq c_{1}{\left(m\;n\right)}^{1-1/d}\left(1-\gamma^{1/d}\right). \end{array}$$

We know that m,n≥1 and $c_{1}\geq d\;h^{d-1}c_{2}$, so it is sufficient to get

(A101) $$\begin{array}{c} c_{2}\left(h^{d-1}-\gamma \right)\leq d\;h^{d-1}c_{2}\left(1-\gamma^{1/d}\right), \end{array}$$

choose *t* as $\gamma =t\;h^{d}$, then 0<t≤1, so (A101) becomes

(A102) $$\begin{array}{c} \left(h^{-1}-t\right)\geq d\;h^{-1}(1-h\;t^{1/d}). \end{array}$$

Note that the function $d\;h^{-1}(1-h\;t^{1/d})+t-h^{-1}$ has a minimum at t=1 which implies (A101) and subsequently (A95). Hence, the proof is completed. □

Lemma A10: (Smoothness for $R_{m,n}$) Given observations of

$$X_{m}:=\left(X_{m^{\prime }},X_{m^{\prime \prime }}\right)=\left\{X_{1},\cdots ,X_{m^{\prime }},X_{m^{\prime }+1},\cdots ,X_{m}\right\},$$

such that $m^{\prime }+m^{\prime \prime }=m$ and $Y_{n}:=\left(Y_{n^{\prime }},Y_{n^{\prime \prime }}\right)=\left\{Y_{1},\cdots ,Y_{n^{\prime }},Y_{n^{\prime }+1},\cdots ,Y_{n}\right\}$, where $n^{\prime }+n^{\prime \prime }=n$, denote $R_{m,n}\left(X_{m},Y_{n}\right)$ as before, the number of edges of $\mathrm{MST}(X_{m},Y_{n})$ which connect a point of $X_{m}$ to a point of $Y_{n}$. Then, for integer h≥2, for all $\left(X_{n},Y_{m}\right)\in {\left[0,1\right]}^{d}$, $\epsilon \geq h^{2}\;\delta_{m,n}^{h}$, where $\delta_{m,n}^{h}=O\left(h^{d-1}{\left(m+n\right)}^{1/d}\right)$, we have

(A103) $$\begin{array}{c} P\left(|R_{m,n}\left(X_{m},Y_{n}\right)-R_{m^{\prime },n^{\prime }}\left(X_{m^{\prime }},Y_{n^{\prime }}\right)|\leq {\tilde{c}}_{\epsilon ,h}\;{\left(\#X_{m^{\prime \prime }}\;\#Y_{n^{\prime \prime }}\right)}^{1-1/d}\right)\geq 1-\frac{2h\;\delta_{m,n}^{h}}{\epsilon }, \end{array}$$

where ${\tilde{c}}_{\epsilon ,h}=O\left(\frac{\epsilon }{h^{d-1}-1}\right)$. For the case $\epsilon <h^{2}\;\delta_{m,n}^{h}$, this holds trivially.

**Proof.** We begin with removing the edges which contain a vertex in $X_{m^{\prime \prime }}$ and $Y_{n^{\prime \prime }}$ in minimal spanning tree on $\left(X_{m},Y_{n}\right)$. Now, since each vertex has bounded degree, say $c_{d}$, we can generate a subgraph in which has at most $c_{d}\left(\#X_{m^{\prime \prime }}+\#Y_{n^{\prime \prime }}\right)$ components. Next, choose one vertex from each component and form the minimal spanning tree on these vertices, assuming all of them can be considered in FR test statistic, we can write

(A104) $$\begin{array}{c} \begin{array}{c} R_{m,n}\left(X_{m},Y_{n}\right)\leq R_{m^{\prime },n^{\prime }}\left(X_{m^{\prime }},Y_{n^{\prime }}\right)+c_{\epsilon ,h}^{\prime \prime }{\left(c_{d}^{2}\;\#X_{m^{\prime \prime }}\;\#Y_{n^{\prime \prime }}\right)}^{1-1/d},\\ \mathrm{or}\;\mathrm{equivalently}\\ \;\;\;\leq R_{m^{\prime },n^{\prime }}\left(X_{m^{\prime }},Y_{n^{\prime }}\right)+c_{\epsilon 1}^{h}{\left(\;\#X_{m^{\prime \prime }}\;\#Y_{n^{\prime \prime }}\right)}^{1-1/d}, \end{array} \end{array}$$

with probability at least g(ϵ), where g(ϵ) is as in Lemma A9. Note that this expression is obtained from Lemma A9. In this stage, it remains to show that with at least probability g(ϵ)

(A105) $$\begin{array}{c} R_{m,n}\left(X_{m},Y_{n}\right)\geq R_{m^{\prime },n^{\prime }}\left(X_{m^{\prime }},Y_{n^{\prime }}\right)-{\tilde{c}}_{\epsilon ,h}\;{\left(\#X_{m^{\prime \prime }}\;\#Y_{n^{\prime \prime }}\right)}^{1-1/d}, \end{array}$$

which, again by using the method before, with at least probability g(ϵ), one derives

$$\begin{array}{c} \begin{array}{c} R_{m^{\prime },n^{\prime }}\left(X_{m^{\prime }},Y_{n^{\prime }}\right)\leq R_{m,n}\left(X_{m},Y_{n}\right)+{\hat{c}}_{\epsilon ,h}\;{\left(c_{d}^{2}\;\left(\#X_{m^{\prime \prime }}\;\#Y_{n^{\prime \prime }}\right)\right)}^{1-1/d},\\ orequivalently\\ \;\;\;\leq R_{m,n}\left(X_{m},Y_{n}\right)+c_{\epsilon 2}^{h}\;{\left(\#X_{m^{\prime \prime }}\;\#Y_{n^{\prime \prime }}\right)}^{1-1/d}. \end{array} \end{array}$$

Letting ${\tilde{c}}_{\epsilon ,h}=\max\left\{c_{\epsilon 1}^{h},c_{\epsilon 2}^{h}\right\}$ implies (A105). Thus,

(A106) $$\begin{array}{c} P\left(|R_{m,n}\left(X_{m},Y_{n}\right)-R_{m^{\prime },n^{\prime }}\left(X_{m^{\prime }},Y_{n^{\prime }}\right)|\geq {\tilde{c}}_{\epsilon ,h}\;{\left(\#X_{m^{\prime \prime }}\;\#Y_{n^{\prime \prime }}\right)}^{1-1/d}\right)\leq 2-2\;g\left(\epsilon \right), \end{array}$$

Hence, the smoothness is given with at least probability 2g(ϵ)−1 as in the statement of Lemma A10. □

Lemma A11: (Semi-Isoperimetry) Let μ be a measure on ${\left[0,1\right]}^{d}$; $\mu^{n}$ denotes the product measure on space ${\left({\left[0,1\right]}^{d}\right)}^{n}$. In addition, let $M_{e}$ denotes a median of $R_{m,n}$. Set

(A107) $$\begin{array}{c} A:=\left\{X_{m}\in {\left({\left[0,1\right]}^{d}\right)}^{m},Y_{n}\in {\left({\left[0,1\right]}^{d}\right)}^{n};R_{m,n}\left(X_{m},Y_{n}\right)\leq M_{e}\right\}. \end{array}$$

Then,

(A108) $$\begin{array}{c} \mu^{m+n}\left(\left\{x^{\prime }\in {\left({\left[0,1\right]}^{d}\right)}^{m},y^{\prime }\in \left({\left[0,1\right]}^{n}\right):\phi_{A}\left(x^{\prime }\right)\;\phi_{A}\left(y^{\prime }\right)\geq t\right\}\right)\leq 4\exp\left(\frac{-t}{8(m+n)}\right). \end{array}$$

**Proof.** Let $\phi_{A}\left(z^{\prime }\right)=\min\left\{H\left(z,z^{\prime }\right),z\in A\right\}$. Using Proposition 6.5 in [17], isoperimetric inequality, we have

(A109) $$\mu^{m+n}\left(\left\{z^{\prime }\in {\left({\left[0,1\right]}^{d}\right)}^{m+n}:\phi_{A}\left(z^{\prime }\right)\geq t\right\}\right)\leq 4\exp\left(\frac{-t^{2}}{8(m+n)}\right).$$

Furthermore, we know that

$${\left(\phi_{A}\left(x^{\prime }\right)+\phi_{A}\left(y^{\prime }\right)\right)}^{2}\geq \phi_{A}\left(x^{\prime }\right)\;\phi_{A}\left(y^{\prime }\right),$$

hence

(A110) $$\begin{array}{c} \mu^{m+n}\left(\left\{(x^{\prime }\in {\left({\left[0,1\right]}^{d}\right)}^{m},y^{\prime }\in \left({\left[0,1\right]}^{n}\right):\phi_{A}\left(x^{\prime }\right)\phi_{A}\left(y^{\prime }\right)\geq t\right\}\right)\\ \;\leq \mu^{m+n}\left(\left\{(x^{\prime }\in {\left({\left[0,1\right]}^{d}\right)}^{m},y^{\prime }\in \left({\left[0,1\right]}^{n}\right):{\left(\phi_{A}\left(x^{\prime }\right)+\phi_{A}\left(y^{\prime }\right)\right)}^{2}\geq t\right\}\right)\\ \;\;=\mu^{m+n}\left(\left\{(x^{\prime }\in {\left({\left[0,1\right]}^{d}\right)}^{m},y^{\prime }\in \left({\left[0,1\right]}^{n}\right):\phi_{A}\left(x^{\prime }\right)+\phi_{A}\left(y^{\prime }\right)\geq \sqrt{t}\right\}\right). \end{array}$$

The last equality in (A110) achieves because of $\phi_{A}\left(x^{\prime }\right),\phi_{A}\left(y^{\prime }\right)\geq 0$ and note that $\phi_{A}\left(z^{\prime }\right)\geq \phi_{A}\left(x^{\prime }\right)+\phi_{A}\left(y^{\prime }\right)$. Therefore,

$$\begin{array}{c} \begin{array}{c} \mu^{m+n}\left(\left\{(x^{\prime }\in {\left({\left[0,1\right]}^{d}\right)}^{m},y^{\prime }\in \left({\left[0,1\right]}^{n}\right):\phi_{A}\left(x^{\prime }\right)+\phi_{A}\left(y^{\prime }\right)\geq \sqrt{t}\right\}\right)\\ \;\;\leq \mu^{m+n}\left(\left\{(z^{\prime }\in {\left({\left[0,1\right]}^{d}\right)}^{m+n}:\phi_{A}\left(z^{\prime }\right)\geq \sqrt{t}\right\}\right). \end{array} \end{array}$$

By recalling (A109), we derive the bound (A108). □

Lemma A12: (Deviation of the Mean and Median) Consider $M_{e}$ as a median of $R_{m,n}$. Then, for given $g\left(\epsilon \right)=1-\frac{h\;\delta_{m,n}^{h}}{\epsilon }$, and $\delta_{m,n}^{h}=O\left(h^{d-1}{\left(m+n\right)}^{1/d}\right)$ such that for h≥7, $\epsilon \geq h^{2}\delta_{m,n}^{h}$, we have

(A111) $$|E\left[R_{m,n}\left(X_{m},Y_{n}\right)\right]-M_{e}|\leq C_{m,n}\left(\epsilon ,h\right)\;{\left(m+n\right)}^{(d-1)/d},$$

where $C_{m,n}\left(\epsilon ,h\right)$ stands with a form depends on ϵ, *h*, *m*, *n* as

(A112) $$\begin{array}{c} C_{m,n}\left(\epsilon ,h\right)=C\;{\left(1-\left({\left(2\;{\left(2\;g\left(\epsilon \right)-1\right)}^{2}\right)}^{-1}\right)\right)}^{-1}, \end{array}$$

where *C* is a constant.

**Proof.** Following the analogous arguments in [17,53], we have

(A113) $$\begin{array}{c} |E\left[R_{m,n}\left(X_{m},Y_{n}\right)\right]-M_{e}|\leq E|R_{m,n}\left(X_{m},Y_{n}\right)-M_{e}|=\int_{0}^{\infty }P\left(|R_{m,n}\left(X_{m},Y_{n}\right)-M_{e}|\geq t\right)\;dt\\ \;\leq 8\;{\left(1-\left(1/\left(2\;{\left(2\;g\left(\epsilon \right)-1\right)}^{2}\right)\right)\right)}^{-1}\int_{0}^{\infty }\exp\left(\frac{-t^{d/(d-1)}}{8{\left(4\epsilon \right)}^{d/d-1}\left(m+n\right)}\right)\;dt\\ \;\;=C\;{\left(1-\left({\left(2\;{\left(2\;g\left(\epsilon \right)-1\right)}^{2}\right)}^{-1}\right)\right)}^{-1}\;{\left(m+n\right)}^{(d-1)/d}, \end{array}$$

where $g\left(\epsilon \right)=1-\left(h\;O\left(h^{d-1}{\left(m+n\right)}^{1/d}\right)\right)/\epsilon$. The inequality in (A113) is implied from Theorem 5. Hence, the proof is completed. □
